# Interleukin-1 receptor-associated kinase 4 (IRAK4) is a critical regulator of inflammatory signalling through toll-like receptors 4 and 7/8 in murine and human lungs

**DOI:** 10.1111/bph.16509

**Published:** 2024-08-13

**Authors:** Ian Sayers, Dhruma Thakker, Charlotte Billington, Stefan Kreideweiss, Marc A. Grundl, Thierry Bouyssou, Sven Thamm, Sebastian Kreuz, Ian P. Hall

**Affiliations:** 1 Centre for Respiratory Research, NIHR Nottingham Biomedical Research Centre, School of Medicine, Biodiscovery Institute, University of Nottingham, Nottingham, UK; 2 Boehringer Ingelheim Pharma GmbH & Co. KG, Biberach, Germany

**Keywords:** chronic obstructive pulmonary disease, exacerbations, interstitial lung disease, IRAK4, lung inflammation, toll- like receptors

## Abstract

**Background and Purpose:**

Toll-like receptors 4 (TLR4) and TLR7/TLR8 play an important role in mediating the inflammatory effects of bacterial and viral pathogens. Interleukin-1 receptor-associated kinase 4 (IRAK4) is an important regulator of signalling by toll-like receptor (TLR) and hence is a potential therapeutic target in diseases characterized by increased lung inflammatory signalling.

**Experimental Approach:**

We used an established murine model of acute lung inflammation, and studied human lung tissue ex vivo, to investigate the effects of inhibiting IRAK4 on lung inflammatory pathways.

**Key Results:**

We show that TLR4 stimulation produces an inflammatory response characterized by neutrophil influx and tumour necrosis factor-α (TNF-α) production in murine lungs and that these responses are markedly reduced in IRAK4 kinase-dead mice. In addition, we characterize a novel selective IRAK4 inhibitor, BI1543673, and show that this compound can reduce lipopolysaccharide (LPS)-induced airway inflammation in wild-type mice. Additionally, BI1543673 reduced inflammatory responses to both TLR4 and TLR7/8 stimulation in human lung tissue studied ex vivo.

**Conclusion and Implications:**

These data demonstrate a key role for IRAK4 signalling in lung inflammation and suggest that IRAK4 inhibition has potential utility to treat lung diseases characterized by inflammatory responses driven through TLR4 and TLR7/8.

## Abbreviations

COPD: chronic obstructive pulmonary disease
ILD: interstitial lung disease
IL: interleukin
IRAK: interleukin 1 receptor associated kinase
TNF: tumour necrosis factor
TLR: toll-like receptor

## 1 Introduction

Lung inflammation plays a key role in the development and progression of many airway diseases including asthma, chronic obstructive pulmonary disease (COPD) and interstitial lung diseases (ILDs). Inflammatory signalling can be triggered through a range of mechanisms, but one of the more important signalling pathways in the lung involves stimulation of toll-like receptors (TLRs) by mediators released by pathogens and other environmental stimuli. TLRs are pattern recognition receptors, and products of bacterial and viral pathogens act via TLRs to induce receptor dimerization, which in turn causes aggregation of their intracellular toll–interleukin receptor (TIR) domains. Bacterial lipopolysaccharide (LPS) predominantly activates toll-like receptor 4 (TLR4), but viral stimuli preferentially engage TLR7 / TLR8. TLR activation then acts as a platform for signalling via recruitment of the adaptor proteins MyD88 and TIR domain-containing adaptor protein (TIRAP) and recruitment of kinases, namely interleukin-1 receptor-associated kinase 1 (IRAK1) and interleukin-1 receptor-associated kinase 4 (IRAK4), through death domain interactions. This can then lead to further recruitment of IRAK1 and interleukin-1 receptor-associated kinase 2 (IRAK2) and additional downstream signalling.

We have previously shown that LPS, a known agonist at TLR4, produces an inflammatory response in human lung tissue explants (Rimington et al., 2017) and that this response is characterized by the production of a range of cytokines and mediators including interleukin-8 (IL-8), interleukin-6 (IL-6), interleukin-1β (IL-1β) and tumour necrosis factor-α (TNF-α). TLR7/8 stimulation also is known to be pro-inflammatory in a range of preclinical lung models (Huang et al., 2022; Rappe et al., 2021; Salvi et al., 2021) but has not previously been studied in the human lung explant system we have previously characterized. Given the key role of IRAK4 in downstream inflammatory signalling from TLR4, TLR7 and TLR8, the aim of the current study was to evaluate whether selective inhibition of IRAK4 could reduce lung inflammatory responses to TLR agonists active at either TLR4 or TLR7/8.

To investigate the role of IRAK4, we initially used transgenic mouse models expressing a kinase-dead IRAK4 mutant to further define the role of IRAK4 kinase activity in murine lung inflammation induced by LPS. We also synthesized a novel IRAK4 inhibitor, BI1543673, to help investigate the role of IRAK4 in inflammatory signalling following TLR4 and TLR7/8 stimulation in a range of models relevant to airway disease including murine lung models, murine blood and ex vivo human lung fragments. Here, we report that IRAK4 plays a key role in regulating lung inflammation driven by TLR4 and TLR7/8 stimulation and that a selective inhibitor of IRAK4 can reduce inflammatory signalling in both murine and human lungs.

## 2 Methods

### 2.1 Study design

A range of in vitro and in vivo methods were used in this study as detailed below. Animal studies are reported in compliance with the ARRIVE guidelines (Percie du Sert et al., 2020) and with the recommendations made by the *British Journal of Pharmacology* (Lilley et al., 2020).

### 2.2 LPS-induced lung inflammation model in mice

Adult, test-naive mice (C57BL/6, weighing 18–20 g) were purchased from Charles River Laboratories (Germany). A knock-in mouse strain in which IRAK4 was replaced by the kinase-inactive IRAK4 (D329A) mutant was generated at TaconicArtemis (Germany) as described else-where (Corzo et al., 2020, see also supplementary material). In brief, IRAK4 (D329A) knock-in mice were developed using embryonic stem cells from C57BL/6 mice and were further back-crossed to C57BL6/J mice for six generations. We used these mice to demonstrate that the effects were solely driven by the catalytic activity of IRAK4. Further breeding of this mouse strain was carried out at Charles River Laboratories (Germany). All animals were housed in isolated ventilated cages under a 12-h light–dark cycle and received food and water ad libitum. All animal experimentation was conducted in accordance with German national guidelines and legal regulations. Lung inflammation was induced in C57BL/6 mice by LPS inhalation. Briefly, a 1-mg·ml^-1^ LPS solution (*Escherichia coli* 055:B5, Sigma-Aldrich) was prepared in phosphate-buffered saline (PBS), and the animals were challenged with the nebulized solution for 30 min using the MiniHeart Hi-Flo continuous nebulizer (Westmed). Four hours after the LPS challenge, animals were killed by cervical dislocation, venous blood samples were collected and a lung lavage was performed using 2 × 0.8·ml lavage buffer (Hank's buffered salt solution [HBSS] [HyClone] with 10-U·ml^-1^ heparin). For the LPS challenge experiments, the mice used were females at 8–10 weeks of age.

Cell counts in the lavage fluid and cell differentiation were determined using a haematocytometer (Sysmex XT-2000i), following the suppliers' instructions. TNF-α concentration in the lavage fluid was measured by enzyme-linked immunosorbent assay (ELISA) (OptEIA Mouse TNF-α Set II; Becton Dickinson), following the suppliers’ instructions. For compound testing, BI1543673 was dissolved in 0.5% hydroxyethylcellulose (pH 4). IRAK4 wild-type animals were treated with BI1543673 or vehicle 1 h before LPS challenge. Compound or vehicle was applied orally by gavage (10-ml·kg^−1^ body weight).

### 2.3 Cigarette smoke-induced airway inflammation model

Mice were exposed to the smoke from five regular, non-filter cigarettes (1.2-mg nicotine and 12-mg condensate) a day for 4 days. Cigarette smoke was delivered into the cabinet by passing air at a flow rate of 0.3 ml·s^-1^ through a burning cigarette in a chamber. The combustion time of the cigarette was 16 min. Fresh air was delivered into the cabinet for 8 min to remove the smoke. At intervals of 24 min, the smoke of a new cigarette was delivered into the cabinet. Control animals were treated with ambient air.

Eighteen hours after the last smoke challenge, animals were killed, and a lung lavage was performed using 2 × 0.8-ml lavage buffer (HBSS [HyClone]). Cell counts in the lavage fluid and cell differentiation were determined using a haematocytometer (Sysmex XT-2000i), following the suppliers' instructions. TNF-α concentration in the lavage fluid was measured using the V-PLEX Proinflammatory Panel 1 Mouse Kit (Meso Scale Diagnostics) following the suppliers' instructions.

### 2.4 Structure and synthesis of BI1543673

See the supporting information for full details of the synthesis of BI1543673. For the in vitro assays, BI1543673 was dissolved in dimethyl sulfoxide (DMSO), and serial dilutions were prepared in DMSO prior to final dilution in assay buffer. The final DMSO concentration in all assays was <1%. For animal studies, BI1543673 was dissolved in 0.5% hydroxyethylcellulose (pH 4). All experiments included appropriate DMSO controls based on the final % present.

### 2.5 Activity of BI1543673 on interleukin-1 receptor-associated kinase (IRAK) isoforms in in vitro kinase assays

The kinase domains of human IRAK4 (aa 153–460) and mouse IRAK4 (aa 153–459) were produced as N-terminal His6-tagged proteins in SF9 cells (CLS, Cat No. 604328, RRID:CVCL_0549) and purified by affinity chromatography. In vitro inhibition of IRAK4 activity was measured using the IMAP Fluorescence Polarization Technology (Molecular Devices). Briefly, BI1543673 was dissolved in DMSO and mixed at different concentrations with 1.25-ng human IRAK4 or 2.5-ng murine IRAK4 in IMAP Reaction Buffer (with 0.1% Tween 20 and 1-mM dithiothreitol [DTT]) on 384-well plates. The enzymatic reaction was started with the addition of IRAK4 substrate (5-FAMARFSRFAGSSPSQSSMVAR-OH; Molecular Devices; 100-nM final concentration) and ATP (Promega; 100-μM final concentration). The plates were incubated for 60 min at room temperature in the dark, and the reaction was stopped by the addition of a threefold excess Progressive Binding Reagent (1/400 in binding buffer). The plates were incubated for 30 min at room temperature in the dark, and fluorescence polarization was measured at 485-nm excitation/525-nm emission in an EnVision multilabel reader. Data analysis was performed by calculating the percentage of IRAK4 inhibition in the presence of BI1543673 compared with the vehicle control samples. IC_50_ values were generated by a non-linear regression fit.

### 2.6 Ex vivo whole blood assay in mice

A 600-ng·ml^−1^ (6-μM) LPS (*E. coli* 055:B5, Sigma-Aldrich) was prepared in PBS. An 80-μl venous blood sample was mixed with 100-μl RPMI-1640 medium (Gibco) and 20 μl of 600-ng·ml^−1^ LPS or mixed with 100-μl RPMI-1640 and 20-μl PBS. Blood was anti-coagulated with 1% lithium heparin. The samples were incubated for 4 h at 37°C, and TNF-α concentration was measured in the plasma fraction by ELISA (OptEIA Set Mouse TNF-α, Becton Dickinson), following the suppliers’ instructions.

### 2.7 Human whole blood assay

Venous whole blood was sampled from healthy volunteers who had previously given their informed consent. All samples were anonymized prior to use. Whole blood samples were diluted 1:1 with RPMI-1640 medium (Gibco). BI1543673 was administered to blood samples on 96-well plates at final concentrations (in descending order) of 10 μM, 2 μM, 400 nM, 80 nM, 16 nM, 3.2 nM and 0.64 nM. TNF-α production was stimulated by the addition of 1-μM resiquimod (Invivogen), and the samples were incubated over night at 37°C. As controls, each plate contained samples without stimulation and samples stimulated with resiquimod and without BI1543673. After stimulation, TNF-α concentration was measured in the plasma fractions by ELISA (Human TNF-α ELISA; Becton Dickinson), following the suppliers’ instructions. Data analysis was performed by calculating the TNF-α concentration in the presence of BI1543673 compared to TNF-α without BI1543673 after subtracting the TNF-α level in samples without resiquimod stimulation. IC_50_ values were generated by a non-linear regression fit.

### 2.8 Human lung tissue explant experiments

Human parenchymal lung tissue was obtained from unaffected lung with informed written consent from patients undergoing lung resection surgery, the majority being for suspected malignancy, at either the Royal Papworth or Addenbrooke's Hospital Tissue Bank, UK (REC 18/EE/0269). The majority of donors (11/16) were exsmokers (quit smoking ≥5 years), four individuals were current smokers and one individual was a never smoker. There were nine males and seven females with a mean age of 66.8 ± 10.7 years. Initial analyses of >50 donor samples showed no significant differences in the relevant cytokine responses in individuals based on FEV_1_/FVC values. The tissue sample was first washed in Tyrode's buffer solution, and the whole tissue weight was taken. The tissue sample was then dissected into small pieces of 50–100 mg (wet weight) and incubated for 24 h in 1 ml of RPMI-640 media (with 2.05-mM L-glutamine and 25-mM HEPES) (Sigma, 51536C) containing antibiotics and antimycotics (penicillin, streptomycin and amphotericin B) (Sigma, A5955). After resting overnight, the media were replaced, and explants were stimulated with media, or the TLR4-agonist LPS (Sigma L2654-1MG LPS from *E. coli*) or the TLR7/8-agonist resiquimod. Additional experiments were performed where the IRAK4 inhibitor BI1543673 was added 30 min before the agonist. Tissue samples were incubated for a further 48 h, followed by the collection of supernatants for subsequent protein analyses. Experimental conditions for tissue work in this study were performed in duplicate for each donor.

### 2.9 Luminex assays

We chose a panel of 10 analytes (IL-6, IL-8, TNF-α, monocyte chemoattractant protein-1 [MCP-1/CCL2], IL-1β, macrophage inflammatory protein-1a [MIP1a], macrophage inflammatory protein-1α [MIP1α/CCL3], granulocyte–macrophage colony-stimulating factor [GM-CSF], monocyte chemoattractant protein-4 [MCP-4/CCL13)] and eotaxin-3 [CCL26]) based on previous work to provide a range of outcomes to be assayed in lung explant supernatants (Nanda et al., 2016). A Luminex Screening Assay (with Human Premixed Multi-Analyte Kit, R&D Systems™, Cat No. LXSAHM) was used to analyse tissue culture supernatants as per the manufacturer's instruction. In the Luminex assay plate, each supernatant sample was assayed in duplicate.

### 2.10 Statistical analyses

For LPS-induced lung inflammation in mice, a one-way analysis of variance (ANOVA) with Dunnett's multiple comparisons test (compound treatment) and an unpaired *t*-test (comparison of wild-type vs. kinase-dead mice) were used. For cigarette smoke-induced airway inflammation analyses, an unpaired *t*-test was used. A *P*-value less than 0.05 was considered as significant, and analyses were completed using GraphPad Prism software (Version 7, GraphPad Software Inc.). For in vitro kinase assays, data analysis was performed by calculating the percentage of IRAK4 inhibition in the presence of BI1543673 compared to the vehicle control samples. IC_50_ values were generated by a non-linear regression fit. All cytokine data from explant studies were normalized using wet tissue weights in individual experiments. For dose–response analyses, data were normalized to fold over baseline and EC_50_ value calculated for different cytokines for both LPS and resiquimod. For individual doses and time points, data were normalized to basal levels (100%) and analysed using a one-sample *t*-test and a Wilcoxon test.

For the human lung tissue analyses, n refers to the number of separate donor subjects used for explant studies. The data and statistical analysis comply with the recommendations of the *British Journal of Pharmacology* on experimental design and analysis in pharmacology (Curtis et al., 2022).

### 2.11 Materials

Details of materials and suppliers are provided in specific subsections in Methods.

### 2.12 Nomenclature of targets and ligands

Key protein targets and ligands in this article are hyperlinked to corresponding entries in http://www.guidetopharmacology.org and are permanently archived in the Concise Guide to PHARMACOLOGY 2021/22 (Alexander, Fabbro, Kelly, Mathie, Peters, Veale, Armstrong, Faccenda, Harding, Davies, Beuve, et al., 2023; Alexander, Fabbro, Kelly, Mathie, Peters, Veale, Armstrong, Faccenda, Harding, Davies, Annett, et al., 2023).

## 3 Results

### 3.1 Role of IRAK4 in the regulation of lung inflammation in mice

**Figure 1:**
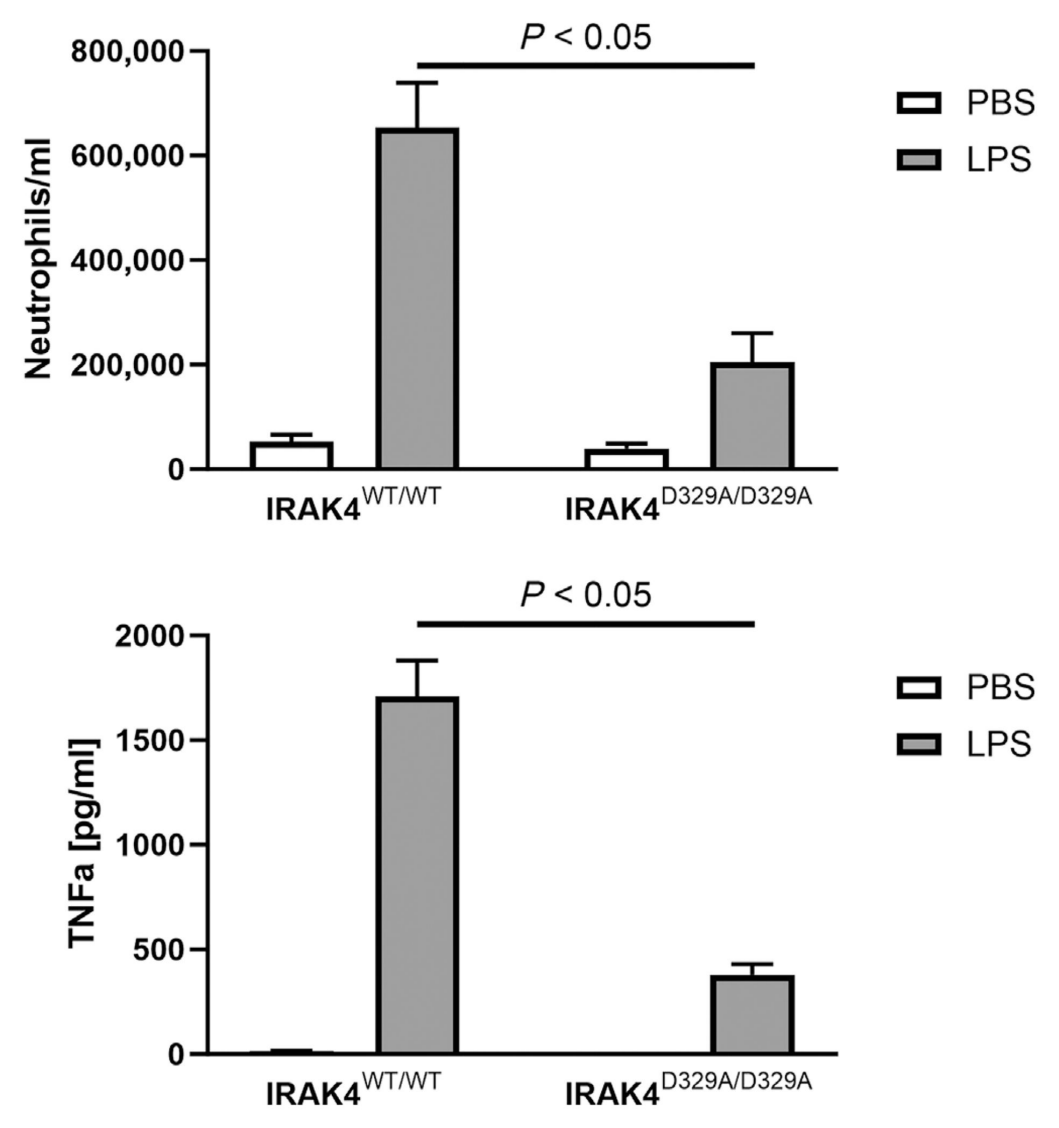
Lipopolysaccharide (LPS)-induced lung inflammation in interleukin-1 receptor-associated kinase 4 (IRAK4) wild-type and IRAK4 kinase-dead mice (IRAK4^D329A/D329A^). LPS or phosphate-buffered saline (PBS) was administered via inhalation, and neutrophil numbers (upper panel) and tumour necrosis factor-α (TNF-α) concentration (lower panel) were measured in bronchoalveolar lavage fluid 4 h after LPS challenge. Data are given as mean ± SEM from 10 animals per group (5 male and 5 female).

To establish proof of concept that selective IRAK4 inhibition could reduce inflammatory signalling in the lung, we first investigated the role of IRAK4 in regulating TLR4-driven lung inflammation using a transgenic mouse in which IRAK4 was replaced by the kinase-inactive IRAK4 D329A mutant. We compared the response of these mice and wild-type animals to lung inflammation induced by inhalation of the TLR4-agonist LPS (see Section 2.2 for details). LPS produced a marked increase in neutrophil numbers and TNF-α levels in bronchoalveolar lavage fluid (BALF) in wild-type mice (Figure 1). Neutrophilic inflammation and TNF-α production were significantly reduced following inhalation with LPS in the IRAK4 kinase-dead mice compared with ‘wild-type’ mice, although baseline levels were not different in the two mouse models (Figure 1). In the kinase-dead mice, neutrophil numbers and TNF-α production were reduced by 73% (*P* < 0.05) and 78% (*P* <0.05), respectively.

**Figure 2:**
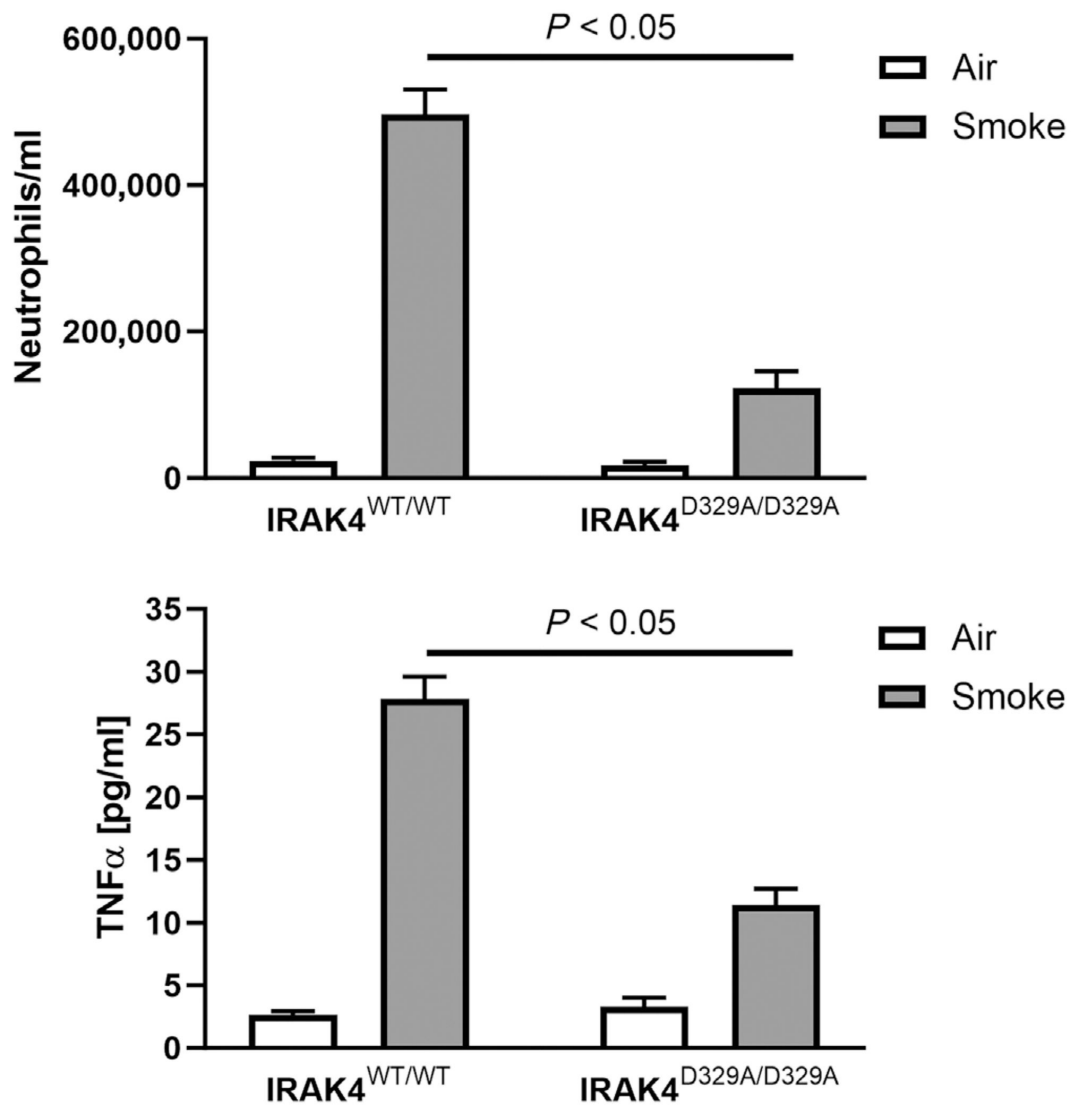
Cigarette smoke-induced lung inflammation in interleukin-1 receptor-associated kinase 4 (IRAK4) wild-type and IRAK4 kinase-dead mice (IRAK4^D329A/D329A^). Animals were challenged with the smoke from five cigarettes a day for four consecutive days, and neutrophil numbers (upper panel) and tumour necrosis factor-α (TNF-α) concentration (lower panel) were measured in bronchoalveolar lavage fluid 18 h after the last smoke treatment. Control animals were treated with ambient air. Data are given as mean ± SEM from five animals per group. Statistics: unpaired *t*-test.

To demonstrate the effect of selective IRAK4 inhibition on lung inflammation triggered by a less specific, disease-relevant stimulus, we characterized the IRAK4 kinase-dead mice in a cigarette smoke exposure model (Figure 2). Mice were exposed to the smoke from five regular, non-filter cigarettes a day for four consecutive days. This challenge produced a marked, neutrophil-driven lung inflammation (characterized by increased neutrophil numbers and TNF-α levels in BALF) in wild-type animals. In the IRAK4 kinase-dead mice, neutrophil numbers and TNF-α production were reduced by 75% (*P* < 0.05) and 68% (*P* < 0.05), respectively (Figure 2). These data establish proof of principle that IRAK4 is important in mediating neutrophilic inflammation in murine lung.

To further characterize the anti-inflammatory effects of BI1543673, we examined other cell types in the BALF; however, the main effects were on reducing numbers and percentage of neutrophils (Tables S2 and S3).

### 3.2 Synthesis of a selective IRAK4 inhibitor, BI1543673, and activity on murine and human IRAK isoforms

To further interrogate the role of IRAK4 in lung inflammation, we designed and synthesized a potent selective IRAK4 inhibitor, BI1543673 (for structure and synthesis, see details in Section 2 and the Methods section of the supporting information). In the Adapta Kinase Profiling Screen (Invitrogen/Thermo Fisher), BI1543673 inhibited only 4 out of 388 tested kinases (including IRAK4 itself) by greater than 50% at a 1-μM concentration (Table S1). BI1543673 is therefore a highly potent inhibitor of the kinase activity of IRAK4. In vitro studies showed that BI1543673 inhibited human and mouse IRAK4 with an IC_50_ value of ~2 nM. TNF-α production in human whole blood stimulated with the TLR7/8-agonist resiquimod was fully inhibited by BI1543673 with an IC_50_ of 26 nM (n = 10).

### 3.3 Inhibition of LPS-induced lung inflammation in mice by BI1543673

**Figure 3:**
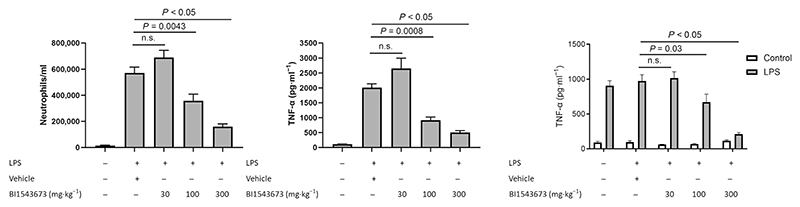
Inhibition of lipopolysaccharide (LPS)-induced lung inflammation by the interleukin-1 receptor-associated kinase 4 (IRAK4) inhibitor BI1543673 in IRAK4 wild-type mice. BI1543673 was administered orally 1 h before challenging the animals by LPS inhalation. Bronchoalveolar lavage fluid (BALF) was prepared 4 h after the LPS challenge, and the neutrophil numbers (left panel) and tumour necrosis factor-α (TNF-α) concentration (centre panel) were measured. Data are given as mean ± SEM from four animals (control group) and eight animals (all other groups). In addition, (right panel) whole blood samples from the animals were stimulated ex vivo with LPS, and the TNF-α production was measured. Data are given as mean ± SEM from two animals (phosphate-buffered saline [PBS]) and six animals (LPS) per group.

Having established a role for IRAK4 in driving inflammation through TLR4 stimulation and proof of principle that a selective inhibitor of IRAK4 is effective in both murine and human in vitro assays, we next used BI1543673 as a tool to investigate the role of IRAK4 in a murine LPS lung inflammation model. Administration of BI1543673 to wild-type mice before LPS challenge resulted in marked inhibition of neutrophil accumulation in BALF, reductions in TNF-α production and reduced TNF-α production from LPS-stimulated whole blood taken from these mice (Figure 3a–c). At the end of these studies, the measured serum levels of BI1543673 in the mice in the 100- and 300-mg·kg^−1^ groups were 560 nM and 2.3 μM, respectively.

### 3.4 TLR4 and TLR7/8 simulation induces cytokine responses in human lung explants ex vivo

**Figure 4:**
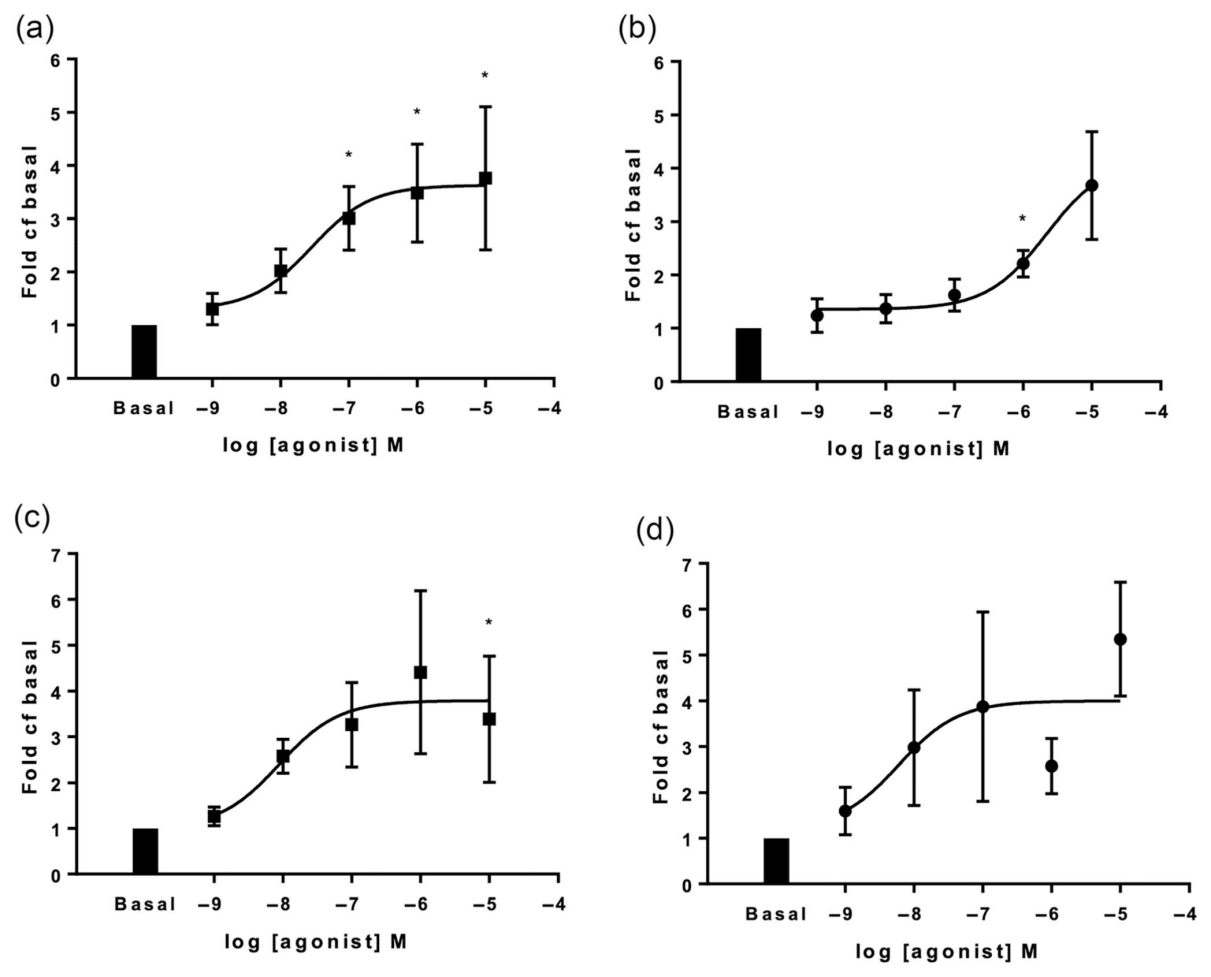
Lipopolysaccharide (LPS) and resiquimod stimulation for 48 h show dose-dependent increases in interleukin-6 (IL-6) and interleukin-8 (IL-8) production in human lung tissue explants. Tissue was stimulated with a range of doses of LPS or resiquimod, and cytokine levels in supernatants were determined by Luminex. (a) LPS-induced IL-8 levels (n = 6), (b) resiquimod-induced IL-8 levels (n = 6), (c) LPS-induced IL-6 levels (n = 7) and (d) resiquimod-induced IL-6 levels (n = 4). Log EC_50_ values were −7.553, −5.664, −8.058 and −8.224, respectively. Data are normalized to basal levels, mean fold ± SEM.

**Figure 5:**
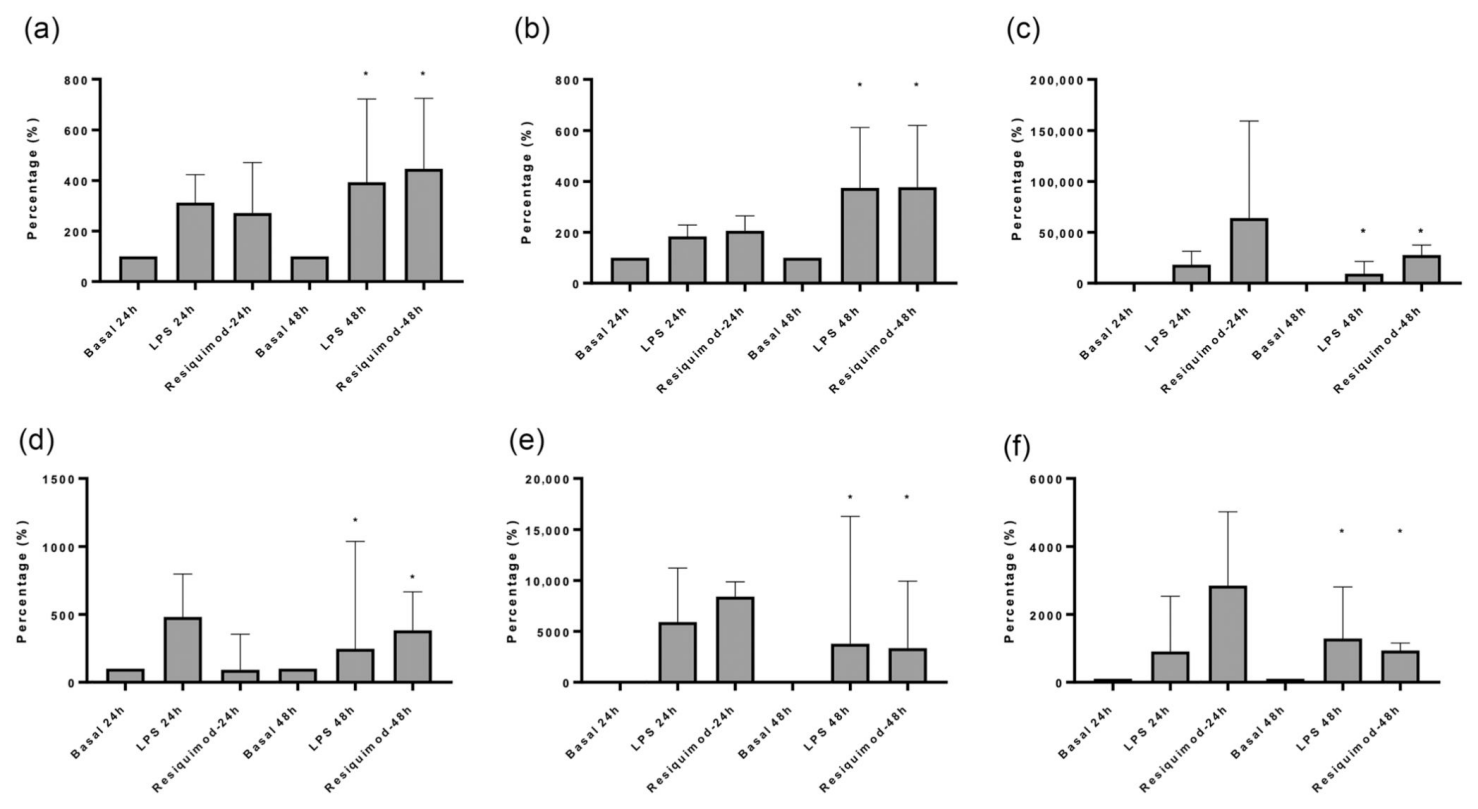
Cytokine production in human lung explants in response to stimulation with lipopolysaccharide (LPS) (10 μM) or resiquimod (10 μM) after either 24- or 48-h stimulations. n = 4–14. (a) interleukin-8 (IL-8), (b) interleukin-6 (IL-6), (c) tumour necrosis factor-α (TNF-α), (d) monocyte chemoattractant protein-1 (MCP-1), (e) interleukin-1β (IL-1β) and (f) macrophage inflammatory protein-1α (MIP1α). Median (interquartile range [IQR]) responses are shown relative to basal (100%).

We have previously demonstrated that the TLR4-agonist LPS induces an inflammatory response in human lung explants (Rimington et al., 2017). In the current study, we also explored the effect on human lung explants of TLR7/8 stimulation using the agonist resiquimod (Kaushik et al., 2021). Both LPS and resiquimod induced dose-dependent cytokine production from explants; examples are shown for release of IL-8 and IL-6 (Figure 4). In further experiments, we observed release of a range of pro-inflammatory cytokines at 48 h in human lung tissue fragments ex vivo (Figure 5). There were some qualitative differences between the responses to TLR4 and TLR7/8 stimulation, with, for example, greater IL-6 and TNF-α responses being observed after stimulation with resiquimod than with LPS (Figure 5). However, taken together, these data demonstrate that activation of both TLR4 and TLR7/8 in the human lung produces a wide-ranging inflammatory response. This is likely to be due to a combination of the release of inflammatory mediators from both in situ inflammatory cells and the airway structural cells present in this tissue.

### 3.5 Inhibition of inflammatory response to TLR4 or TLR7/8 stimulation by the IRAK4 antagonist BI1543673 in human lung tissue

**Figure 6:**
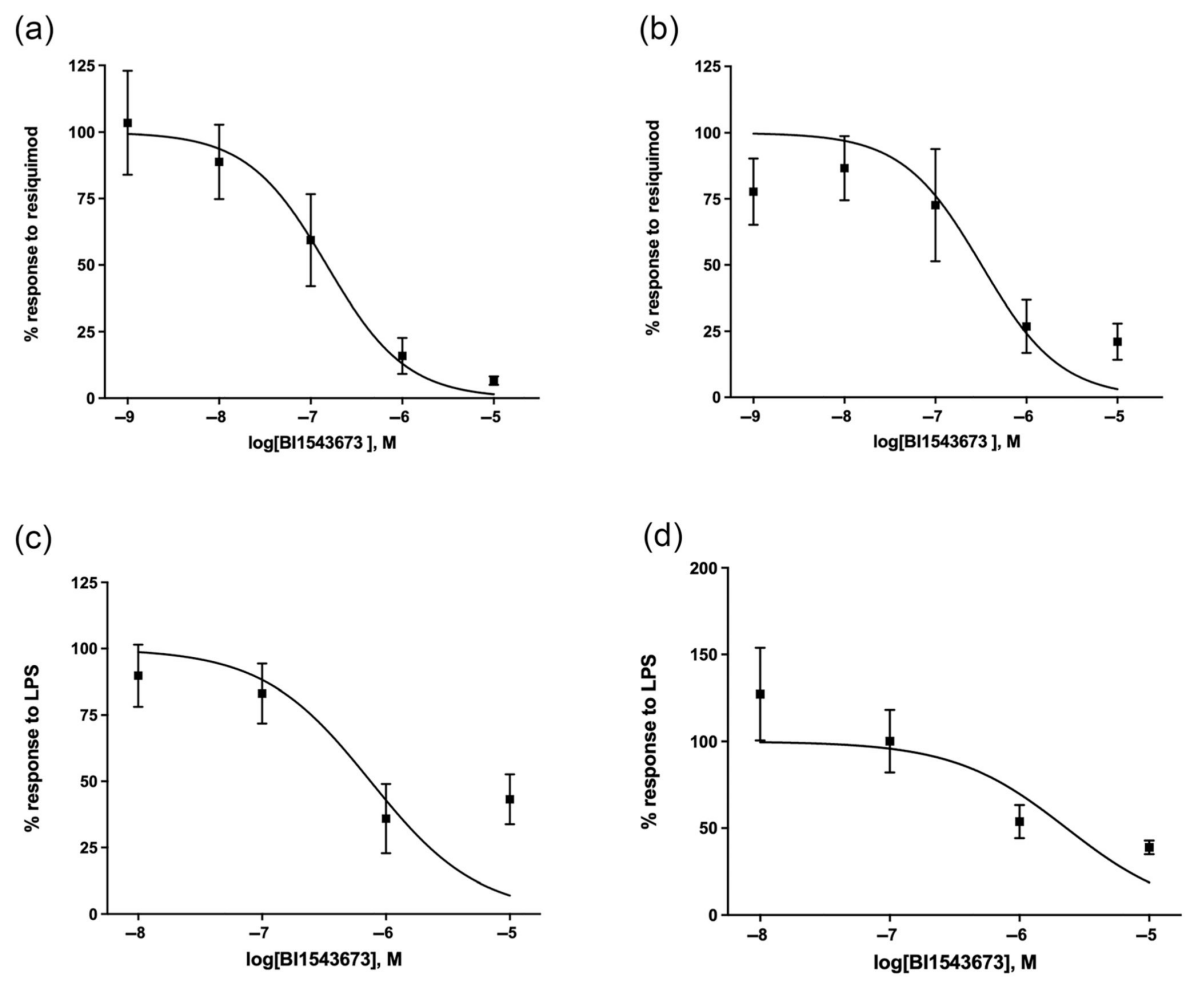
The interleukin-1 receptor-associated kinase 4 (IRAK4) inhibitor BI1543673 significantly inhibits cytokine production in human lung explants in response to resiquimod or lipopolysaccharide (LPS). Tissue was stimulated with 10-μM resiquimod or 10-μM LPS for 48 h ± IRAK4 inhibitor, and cytokine/chemokine levels in supernatants were determined by Luminex. Examples are shown for (a) resiquimod/tumour necrosis factor-α (TNF-α) (n = 6, IC_50_ 0.14 μM), (b) resiquimod/IL-1β (n = 6, IC_50_ 0.32 μM), (c) LPS/IL-1β (n = 6, IC_50_ 0.75 μM) and (d) LPS/granulocyte–macrophage colony-stimulating factor (GM-CSF) (n = 6, IC_50_ 2.30 μM). Data are normalized to the resiquimod or LPS samples for each donor (100% response), mean ± SEM.

To investigate the ability of IRAK4 inhibition to suppress TLR4- or TLR7/8-induced inflammation in human lung tissue, a series of experiments were performed to assess the effects of BI1543673 on inflammatory signalling in human lung tissue fragments ex vivo after stimulation with LPS or resiquimod (both used at 1 μM). Overall, BI1543673 provided robust inhibition across multiple outcomes. Summary IC_50_ values are provided in Table 1 for a range of inflammatory markers released by human lung tissue in response to LPS or resiquimod. Examples of dose-dependent BI1543673-induced inhibition of resiquimod and LPS-stimulated responses are shown for TNF-α and IL-1β (Figure 6a,b) and IL-1β and GM-CSF (Figure 6c,d), respectively.

## 4 Discussion

The data presented in this study show that TLR4 stimulation produces an inflammatory response characterized by neutrophil influx and TNF-α production in murine lungs, and that these responses are markedly reduced in IRAK4 kinase-dead mice. In addition, we have designed and generated a novel selective IRAK4 inhibitor that is able to reduce LPS-induced airway inflammation in wild-type mice. This compound was able to reduce inflammatory responses to both TLR4 and TLR7/8 stimulation in human lung tissue studied ex vivo. Whilst the concentrations of BI1543673 used for the in vivo experiments were higher than the IC_50_ values observed in the in vitro experiments, we chose these concentrations to ensure we achieved at least IC_90_ values of IRAK4 inhibition for the in vivo work. TLR4 is a key signalling pathway in acute lung inflammation (Lowe et al., 2015; Murakami et al., 2007), and TLR7/8 also plays a key role in inflammatory signalling in response to viral infections in the lung including severe influenza and COVID-19 (Rappe et al., 2021; Salvi et al., 2021).

Taken together, therefore, these data suggest that IRAK4 is likely to be a key regulator of inflammatory signalling in acute lung injury driven through TLR4 or TLR7/8 pathways in both murine and human lungs. Further evidence for a key role for IRAK4 in lung inflammation comes from observations in patients homozygous for rare inactivating mutations in IRAK4 and MyD88. These patients are immunocompromised and susceptible to both Gram-negative sepsis and pneumonia, demonstrating the importance of this pathway in inflammatory signalling (von Bernuth et al., 2012). However, this also highlights the potential challenge of targeting IRAK4 clinically to limit acute lung inflammation, because it may be necessary to carefully calibrate inhibition to avoid excessive immunosuppression if IRAK4 inhibitors are to be successfully used in the clinic.

There has been considerable recent interest in the potential clinical use of IRAK4 inhibitors in a range of diseases. Proof-of-principle data exist in preclinical disease models including gout (Kelly et al., 2015), renal failure (Kondo et al., 2014), collagen-induced arthritis (Dudhgaonkar et al., 2017; Kelly et al., 2015; Zarrin & Bao, 2021), lupus (Dudhgaonkar et al., 2017), inflammatory dermatitis (Kelly et al., 2015) and haematological malignancy including activated B cell-like (ABC) subtypes of diffuse large B-cell lymphoma (Corzo et al, 2020; Kelly et al., 2015). However, much less data exist in humans, although two putative IRAK4 inhibitors, BAY-1834845 and Pf-06650833 (Winkler et al., 2021), have recently been studied in phase I clinical trials (see https://clinicaltrials.gov). The former of these agents has been shown to inhibit LPS-induced acute respiratory distress syndrome (ARDS) in a murine model (Li et al., 2022). As well as reducing lung inflammation, BAY-1834845 reduced the expression of genes associated with TLR signalling, as well as TNF-α, interleukin-17 (IL-17) and interferon-induced gene signatures. A recent study on BAY-1834845 enables some further comparisons to be made (Bothe et al., 2024). The IC_50_ value for this compound for IRAK4 inhibition is comparable to that seen with BI1543673 at 8 nM, with 30-fold selectivity over FLT3. In murine studies, BAY-1834845 produced ~80% inhibition of IL-1β-induced IL-6 production at 20 mg-kg^−1^ and around 50% inhibition of IL-1β-induced TNF-α production. At 40 mg-kg^−1^, greater than 80% inhibition of LPS-induced IL-6 or TNF-α production was seen. Very recently, BAY-1834845 has been shown to reduce erythema and cytokine responses to LPS in a human study model using topical imiquimod (Jodl et al., 2024).

Data from a phase 2 study with Pf-06650833 (*Safety and Efficacy of Pf-06650833 In Subjects With Rheumatoid Arthritis, With An Inadequate Response To* Methotrexate) are available at https://clinicaltrials.gov/ct2/show/results/NCT02996500: According to the data presented, the drug was well tolerated and there was no significant increase in the risk of respiratory infections. The IC_50_ value of this compound is ~2 nM, although limited data are available on in vivo effects. In a study in rats in a collagen-induced arthritis model, Pf-06650833 inhibited serum TNF-α production in response to LPS by 87% at a dose of 30 mg-kg^−1^ (Lee et al., 2017). Taken together, therefore, these data suggest that selective IRAK4 inhibitors as a class show promise in inhibiting inflammation in a range of conditions.

For the work presented in this paper, BI1543673 was developed specifically as a tool for exploring IRAK4-driven inflammation, aiming for high potency and good selectivity. Because it has not progressed to date into clinical development, additional pharmacokinetic/ADME data are not currently available.

In summary, given the data presented here together with the other data suggesting key roles for TLR4- and TLR7/8-induced inflammatory signalling in a range of clinically relevant situations, we believe that further evaluation of IRAK4 inhibition as a potential way to reduce lung inflammatory signalling is worthy of consideration. Bacterial and viral inflammation, signalling through TLR4 and TLR7/8, causes exacerbations of a range of different respiratory diseases, including COPD, asthma and ILDs, and plays a key role in the initiation of ARDS (Jain et al., 2023; Jha et al., 2021; Lin et al., 2022). It is therefore likely that inhibition of IRAK4 may be of benefit generally in reducing lung inflammatory signalling. Our data provide proof of concept that the use of IRAK4 inhibitors should be assessed in a broad range of conditions associated with lung-driven inflammation, including COVID-19 pneumonitis.

## Supplementary Material

Additional supporting information can be found online in the Supporting Information section at the end of this article.

Supporting Information

## Figures and Tables

**Table 1:** Efficacy of IRAK4 inhibitor BI1543673 to inhibit the LPS- and/or resiquimod-induced cytokine response in human lung explant tissue.

Analyte	LPS stimulation (IC_50_) (n)	Resiquimod stimulation (IC_50_) (n)
TNF-α	nd	0.14 μM (6)
IL-1β	0.75 μM (6)	0.32 μM (6)
GM-CSF	2.3 μM (6)	5.39 μM (7)
Eotaxin	nd	3.63 μM (6)
IL-8	nd	2.83 μM (6)
IL-6	nd	5.86 μM (7)

Abbreviations: GM-CSF, granulocyte–macrophage colony-stimulating factor; IL-1β, interleukin-1β; IL-6, interleukin-6; IL-8, interleukin-8; IRAK4, interleukin-1 receptor-associated kinase 4; LPS, lipopolysaccharide; nd, not determined; TNF-α, tumour necrosis factor-α.

## Data Availability

The data that support the findings of this study are available on request from the corresponding author. The data are not publicly available due to privacy or ethical restrictions.

## What is already known

- TLR4 and TLR7/8 stimulation induce lung inflammation in preclinical models.

## What does this study add

- IRAK4 inhibition reduces inflammation in murine and human lungs after TLR4 or TLR7/8 stimulation.

## What is the clinical significance

- IRAK4 inhibitors could have clinical value in reducing lung inflammation in exacerbations of lung disease.
