# Supplementary material for: Interleukin-1 receptor-associated kinase 4 (IRAK4) is a critical regulator of inflammatory signalling through toll-like receptors 4 and 7/8 in murine and human lungs
Source: Br J Pharmacol. Author manuscript; Available in PMC 2025 Dec 9. (PMC7618454; doi:10.1111/bph.16509)
Supplement: Supporting Information [file EMS211308-supplement-Supporting_Information.pdf]

Supplementary methods: synthesis and structure of BI154673

Abbreviations:

ACN	acetonitrile
Aq.	Aqueous
°C	Degree celsius
CyH/CH	cyclohexane
conc.	Concentrated
DCM	dichloro methane
DIPEA	*N*,*N*-diisopropylethylamine
DMA	*N*,*N*-dimethylacetamide
DMF	*N*,*N*-dimethylformamide
DMSO	dimethyl sulfoxide
ESI-MS	Electrospray ionisation mass spectrometry
EtOAc	ethyl acetate
EtOH	ethanol
ex	example
eq	equivalent
FA	formic acid
h	hour
HCl	Hydrochlorid acid
HPLC	High performance liquid chromatography
K_2CO_3	potassium carbonate
L	liter

$LiOH*H_2O$	Lithium hydroxide monohydrate
M	molar
2-MeTHF	2-Methyltetrahydrofuran
MeOH	methanol
$MgSO_4$	magnesium sulphate
min	minute
mL	milliliter
MTBE	*tert*-butylmethylether
NH_3	ammonia
Pd(dppf)Cl2	1,1`-Bis(diphenylphosphino)ferrocenedichloropalladium(II)
RT	room temperature (about 20°C)
sat.	saturated
TBTU	Benzotriazolyl tetramethyluronium tetrafluoroborate
TEA	triethylamine
TFA	trifluoroacetic acid
TFAA	trifluoroacetic anhydride
THF	tetrahydrofuran

Preparation of Starting Compounds

Intermediate I

Intermediate I.1

ethyl 6 - methylpyrazolo[1,5 - a]pyrimidine - 3 - carboxylate

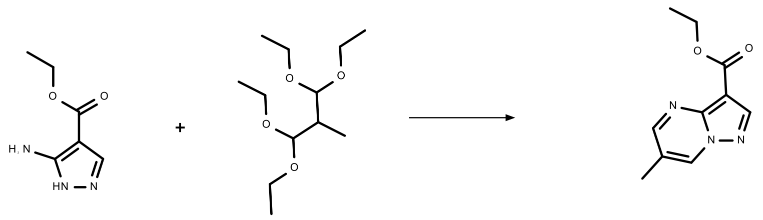

To a solution of 15.5 g (100 mmol) of ethyl 5 - amino - 1H - pyrazole - 4 - carboxylate (Activate) and 23.8 g (101 mmol) 1,1,3,3 - tetraethoxy - 2 - methylpropane (ABCR) in 250 mL ethanol (abs.) was added 27.5 mL (110 mmol) HCL (4 M). The reaction mixture was stirred at 80°C for 24 h. The solvent was removed in vacuo and the residue was diluted with DCM/ water and Na_2CO_3 solution and was extracted. The combined organic phase was dried over Na_2SO_4, filtered and the solvent was removed in vacuo to get 18.9 g (88% yield) of a beige solid.

$C_{10}H_{11}N_3O_2$	(M = 205.2 g/mol)
ESI-MS:	206 $[M+H]^+$
R_t (HPLC):	0.36 min (method A)

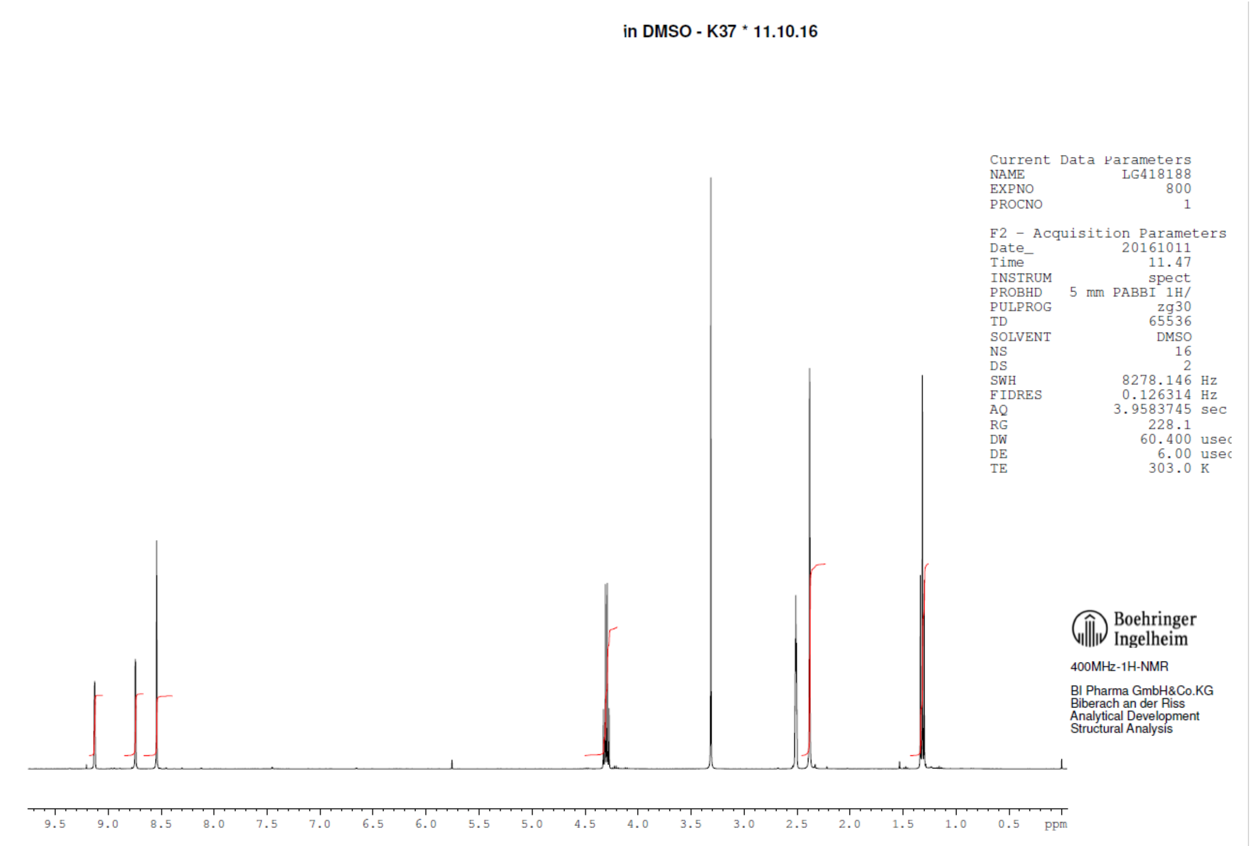

Intermediate I.2

6 - methylpyrazolo[1,5 - a]pyrimidine - 3 - carboxylic acid

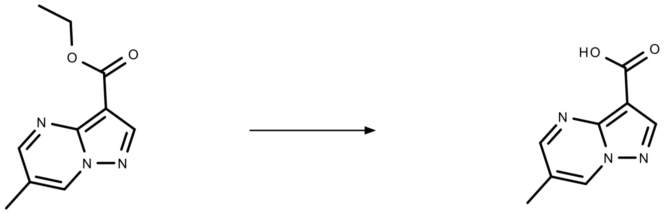

To 18.9 g (82.9 mmol) of ethyl 6 - methylpyrazolo[1,5 - a]pyrimidine - 3 - carboxylate in 33 mL methanol (99.9%) and 66 mL THF (99,9%) was added a suspension of 9.93 g (414 mmol) Lithium hydroxide in 74.6 mL water. The reaction mixture was stirred at 50°C for 2 h. The solvent was removed in vacuo and the residue was acidified with aq. HCl (4M). The precipitation was filtered and washed with water to get 15.1 g (102% yield) of a beige solid.

$C_8H_7N_3O_2$ (M = 177.2 g/mol)

ESI-MS: 178 $[M+H]^+$

R_t (HPLC): 0.24 min (method A)

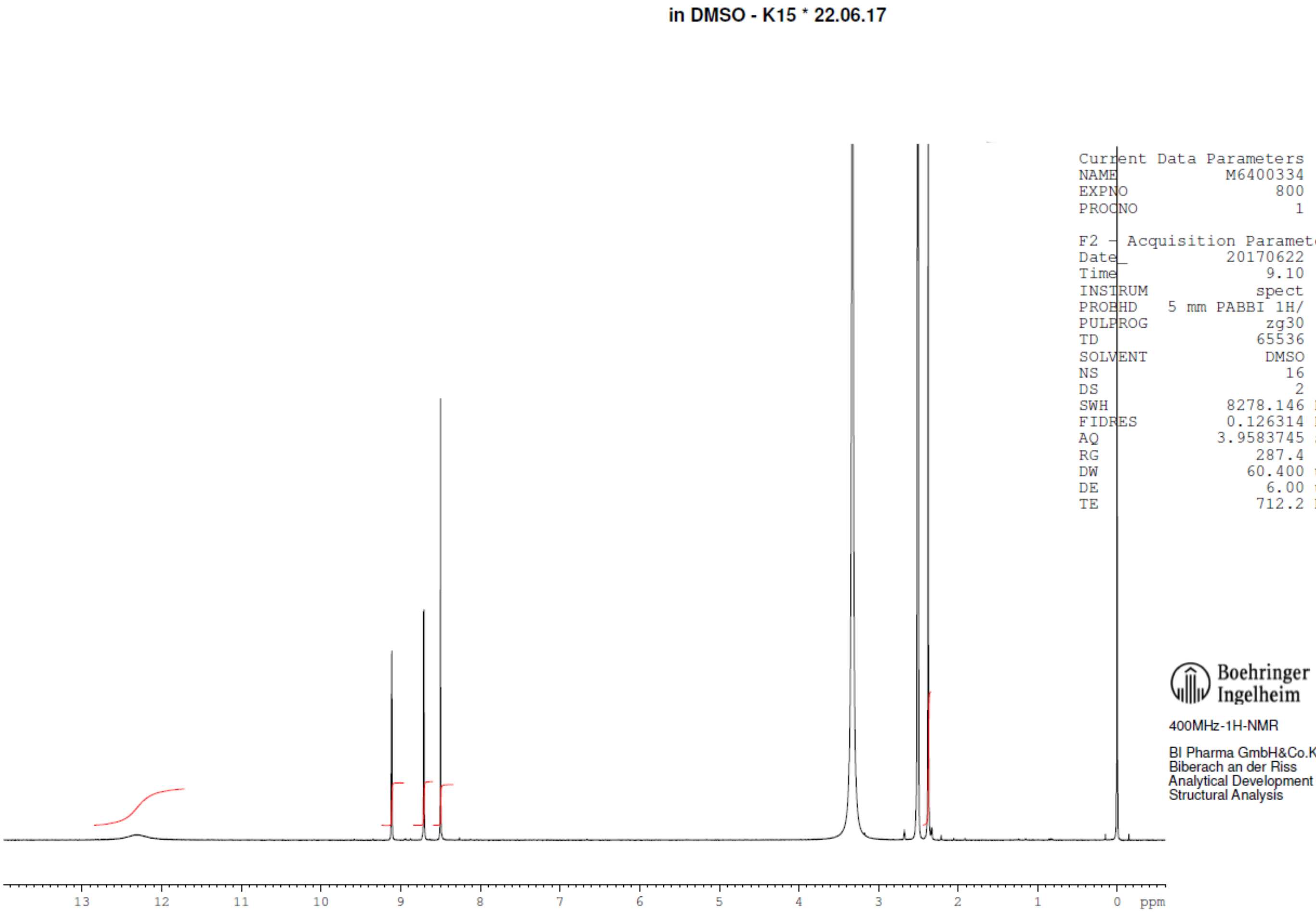

Intermediate II

Intermediate II.1

1 - (2 - amino - 5 - {1,4 - dioxaspiro[4.5]dec - 7 - en - 8 - yl} - 1H - 1,3 - benzodiazol -

1 - yl) - 2 - methylpropan - 2 - ol

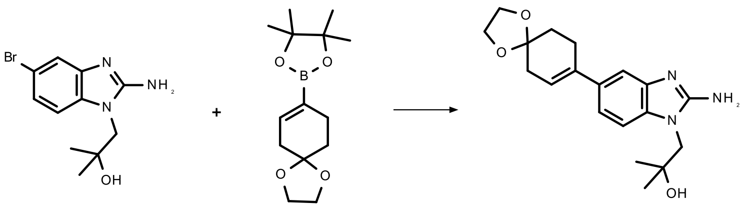

To a argon degassed solution of 40.0 g (140 mmol) 1-(2-amino-5-bromo-1H-1,3-benzodiazol-1-yl)-2-methylpropan-2-ol (TCG), 52.4 g (197 mmol) 2-{1,4-dioxaspiro[4.5]dec-7-en-8-yl}-4,4,5,5-tetramethyl-1,3,2-dioxaborolane (TCG) and 3.45 g Pd(dppf)Cl_2 (4.71 mmol) was added 400 mL dioxane (99,9 %) and 140 mL aq. K_2CO_3-solution (4M). The reaction mixture was stirred under argon at 90°C for 3h. Additional 300 mg (0.41 mmol) of Pd(dppf)Cl_2 were added and the reaction mixture was continued to be stirred under argon at 90°C for 1h, before the reaction mixture was concentrated to about half the volume under reduced pressure. 80 mL water and 240 mL methanol was added at 50°C and was stirred at 50°C. Over 30 min 290 mL water was added and stirred over night at RT. The reaction mixture was cooled with ice water and the precipitation was filtered. 120 mL ACN was added to the crude solid and warmed with stirring to 50°C. 120 mL MTBE was added, then cooled to RT and additional 120 mL MTBE was added. The mixture was cooled to 10°C and stirred for 30 min. The precipitation was filtered and washed with cold MTBE to get 37.7 g (74% yield) of a light brown solid.

$C_{19}H_{25}N_3O_3$	(M = 343.4 g/mol)
ESI-MS:	344 $[M+H]^+$
R_t (HPLC):	0.39 min (method A)

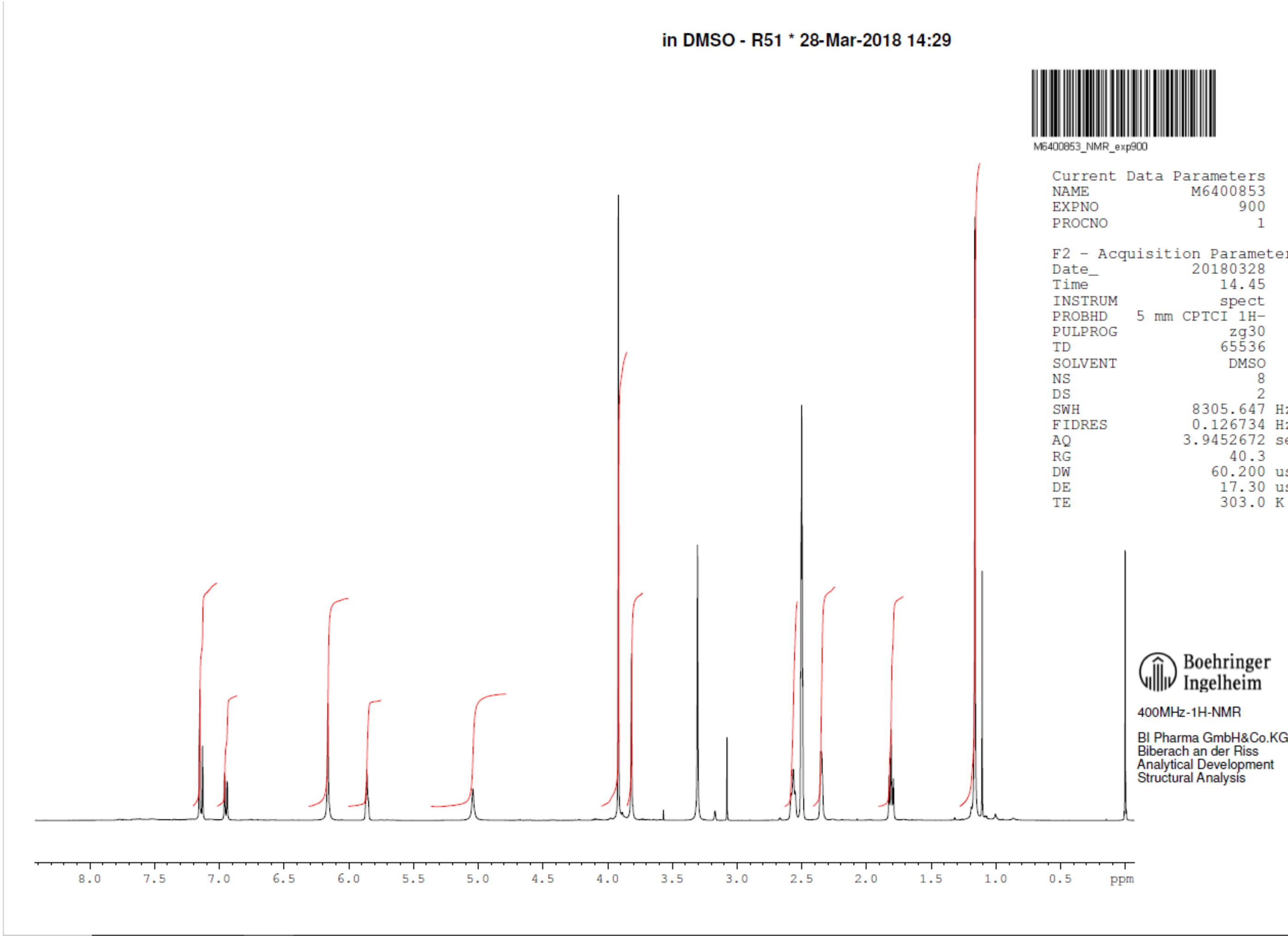

Intermediate II.2

1 - (2 - amino - 5 - {1,4 - dioxaspiro[4.5]decan - 8 - yl} - 1H - 1,3 - benzodiazol - 1 - yl) - 2 - methylpropan - 2 - ol

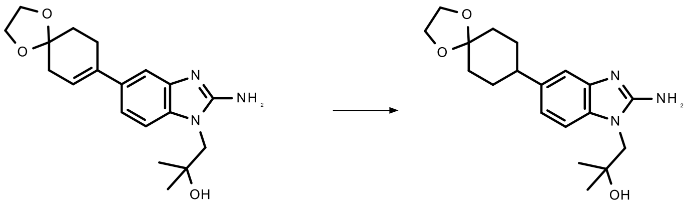

To 37.7 g (110 mmol) 1 - (2 - amino - 5 - {1,4 - dioxaspiro[4.5]dec - 7 - en - 8 - yl} - 1H - 1,3 - benzodiazol - 1 - yl) - 2 - methylpropan - 2 - ol in 3.00 L methanol (99.9%) was added 3.77 g Pd/C. The reaction mixture was hydrogenated at 50°C and 3 bar for 18 h. The

reaction mixture was filtered and concentrated to 120 mL methanol. The residue was diluted with MTBE and heated to 50°C for 30 min. The mixture was slowly cooled to RT and the precipitation was filtered and washed with cold MTBE. The mother liquor was concentrated and the residue was sonicated with 150 mL ACN. The precipitation was filtered and washed with cold MTBE. Both products were combined to get 34.5 g (91% yield) of a sandy tan solid.

$C_{19}H_{27}N_3O_3$	(M = 345.4 g/mol)
ESI-MS:	346 $[M+H]^+$
R_t (HPLC):	0.40 min (method A)

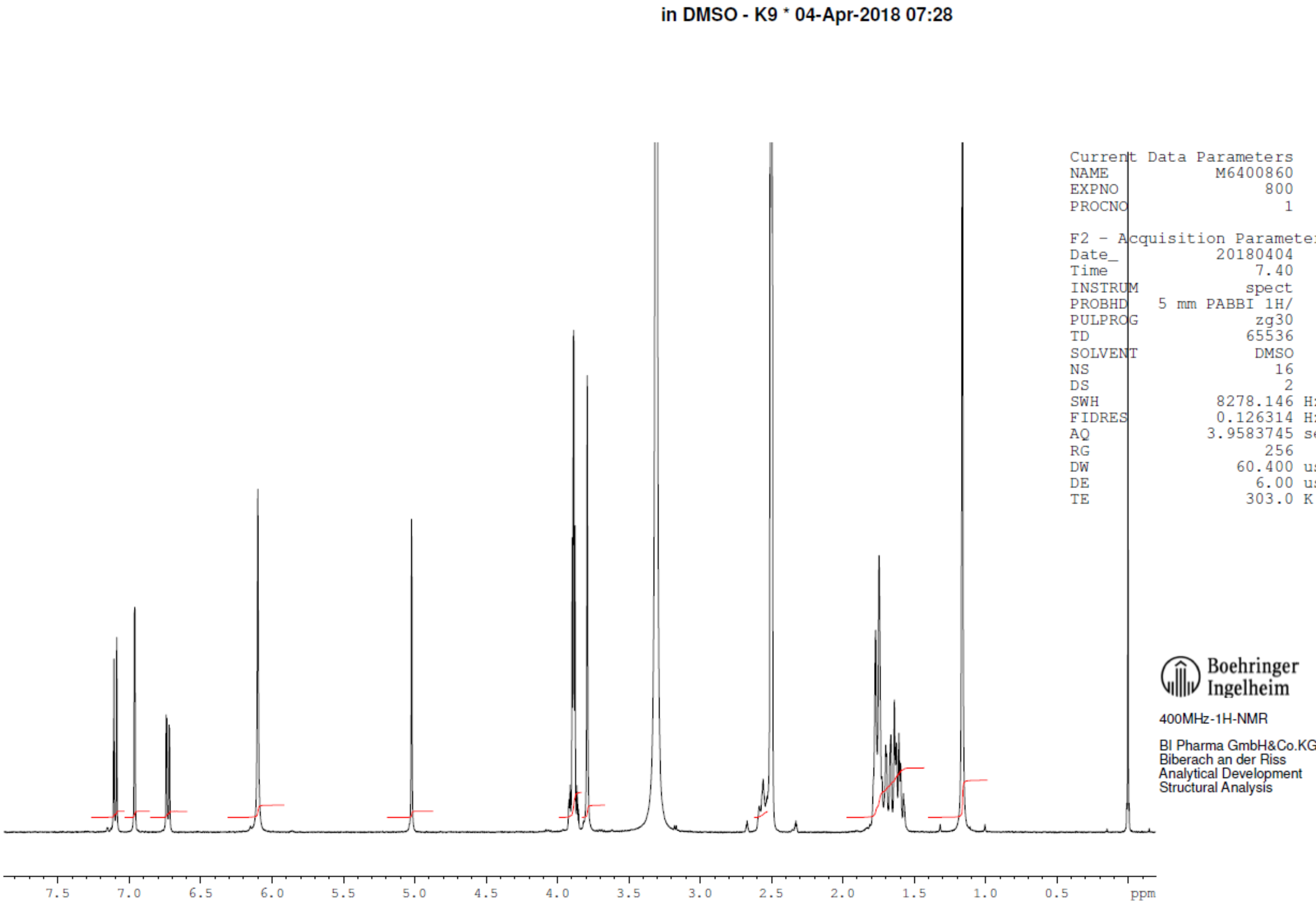

<u>Intermediate II.3</u>

4‐[2‐amino‐1‐(2‐hydroxy‐2‐methylpropyl)‐1H‐1,3‐benzodiazol‐5‐yl]cyclohexan‐1‐one

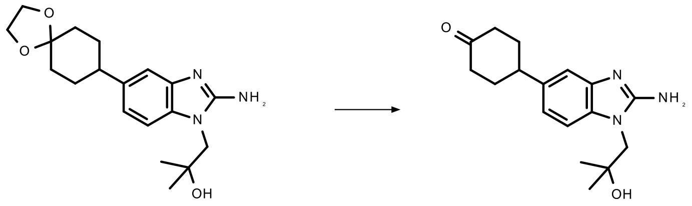

To 34.5 g (99.9 mmol) 1‐(2‐amino‐5‐{1,4‐dioxaspiro[4.5]decan‐8‐yl}‐1H‐1,3‐benzodiazol‐1‐yl)‐2‐methylpropan‐2‐ol in 256 mL THF (99,9%) was added 373 mL 3 N HCl. The reaction mixture was stirred at RT for 90min. The reaction mixture was warmed to 50°C and stirred for 20 min. The reaction mixture was concentrated to half of the THF and basified with 4N NaOH. The reaction mixture was cooled with stirring. Additional water was added and the mixture was cooled for 1h. The precipitation was filtered and washed with water. The product was dried at 50°C over night. The residue was diluted in 2-MeTHF and methanol and was stirred at 40°C. 5 g metal scavenger SiliaMetS ® Thiol bulk material was added and was stirred for 1h. The mixture was filtered over Celite, washed with methanol and concentrated. The residue was dissolved in methanol at 50°C. MTBE was added and sonicated. The precipitation was filtered. The mother liquor was reconcentrated and triturated with ACN and filtered. Both products were combined and dried in the vacuum oven to get 24.9 g (89% yield) of a tan solid.

$C_{17}H_{23}N_3O_2$	(M = 301.4 g/mol)
ESI-MS:	302 $[M+H]^+$
R_t (HPLC):	0.41 min (method B)

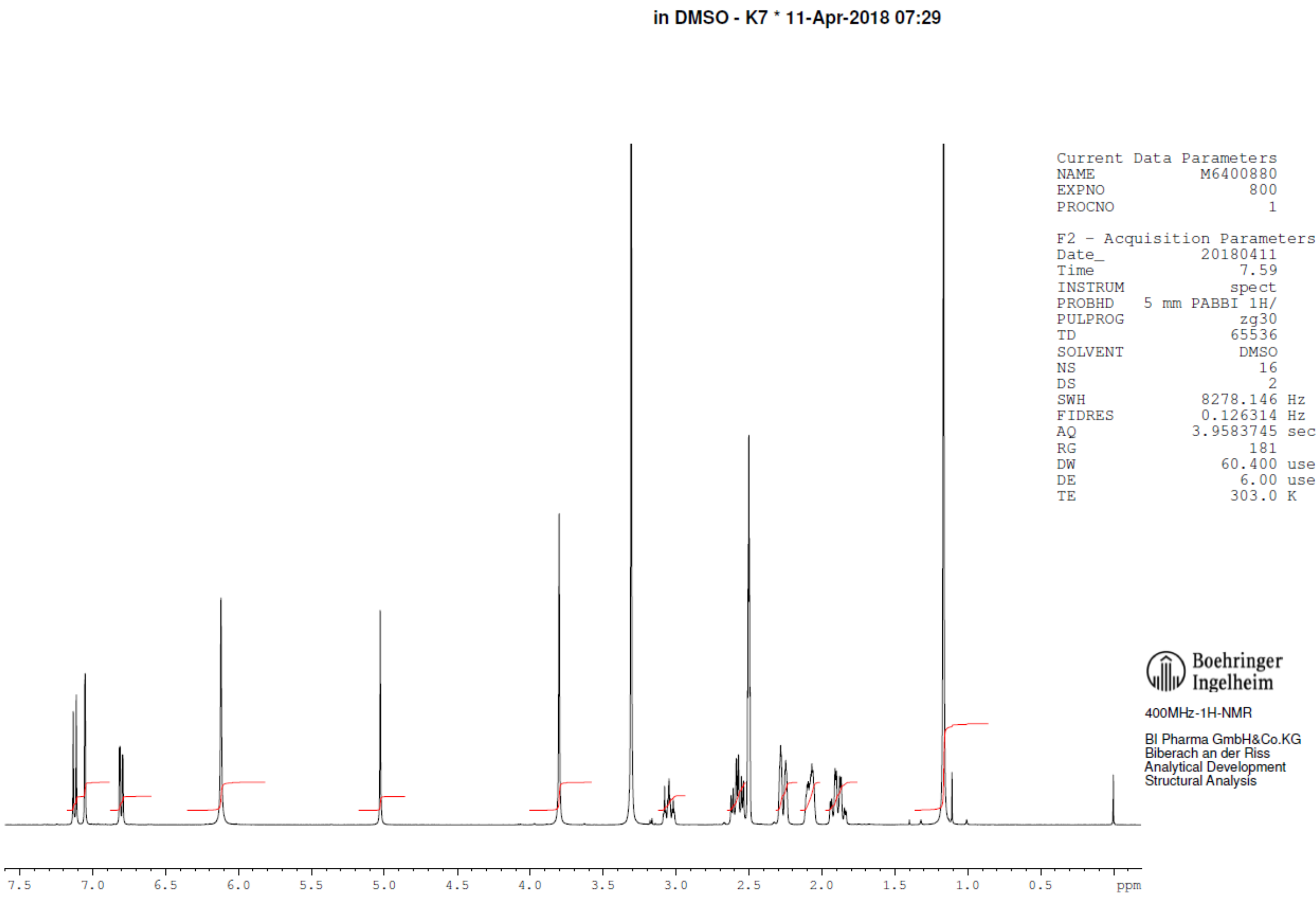

Intermediate II.4

1‐{2‐amino‐5‐[(1s,4s)‐4‐(pyrrolidin‐1‐yl)cyclohexyl]‐1H‐1,3‐benzodiazol‐1‐yl}‐2‐methylpropan‐2‐ol

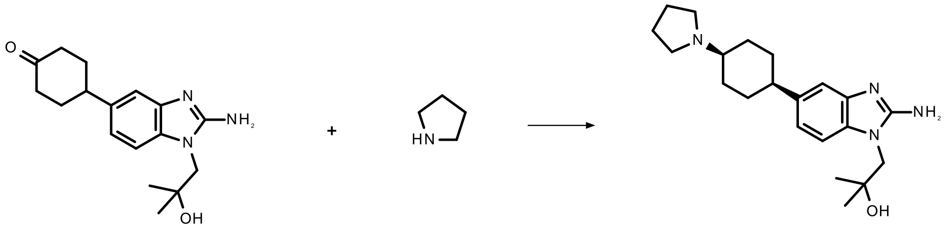

3.00 g (9.95 mmol) 4‐[2‐amino‐1‐(2‐hydroxy‐2‐methylpropyl)‐1H‐1,3‐benzodiazol‐5‐yl]cyclohexan‐1‐one and 1.96 mL (24.9 mmol) Pyrrolidine (Merck) was added 30.0 mL methanol (99.9%) and warmed to 50°C. The mixture was stirred for 1h. To the

reaction mixture was added 300 mg Pd/C and 20 mL methanol. The reaction mixture was hydrogenated at 50°C and 3 bar over night. The reaction mixture was filtered and concentrated under reduced pressure. The residue was purified by HPLC (ACN/H_2O/NH_3) to get 1.93 g (54% yield).

$C_{21}H_{32}N_4O$ (M = 356.5 g/mol)

ESI-MS: 357 $[M+H]^+$

R_t (HPLC): 0.67 min (method D)

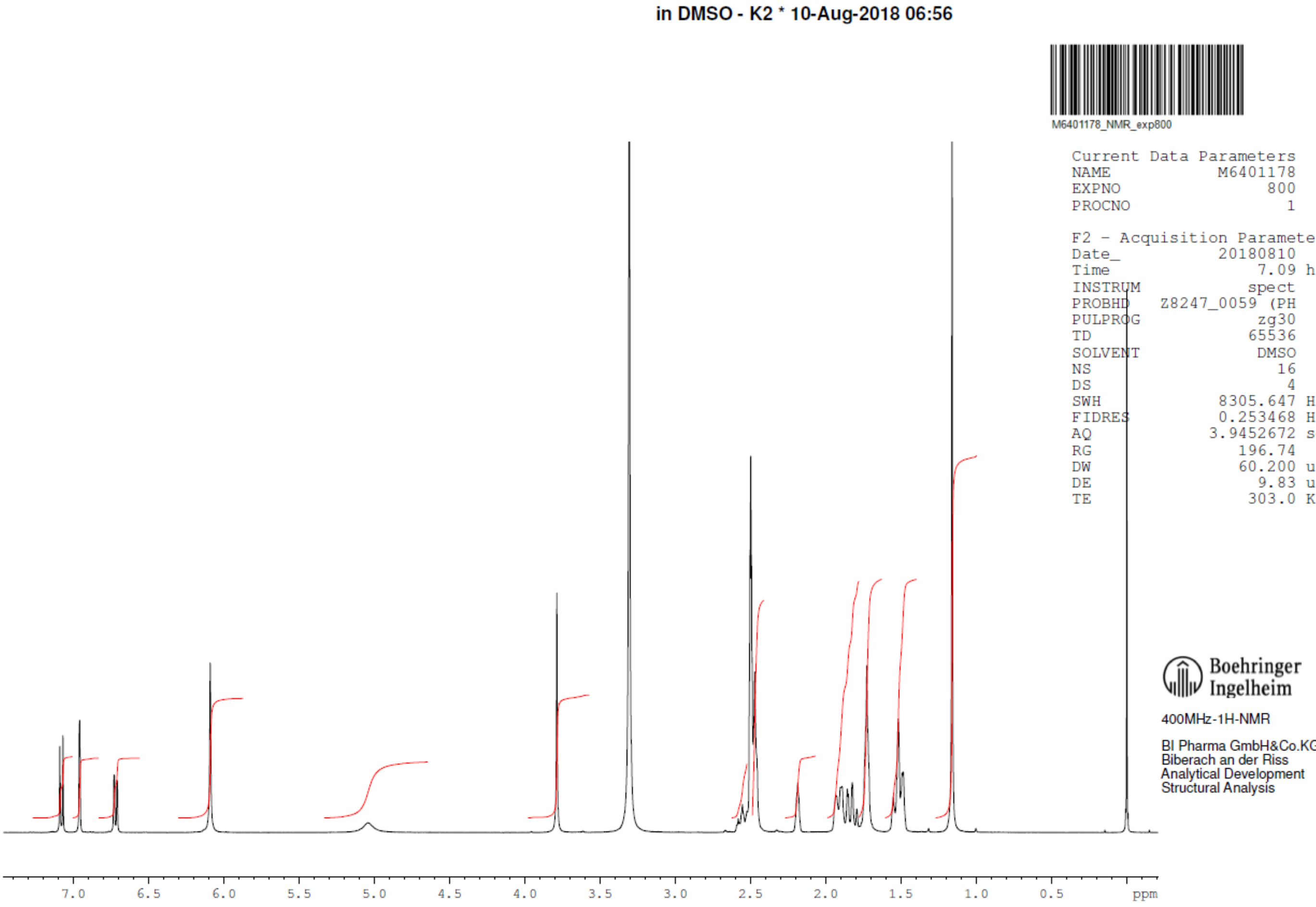

Preparation of Final Compounds

Example 1

N‐[1‐(2‐hydroxy‐2‐methylpropyl)‐5‐[(1s,4s)‐4‐(pyrrolidin‐1‐yl)cyclohexyl]‐1H‐1,3‐benzodiazol‐2‐yl]‐6‐methylpyrazolo[1,5‐a]pyrimidine‐3‐carboxamide

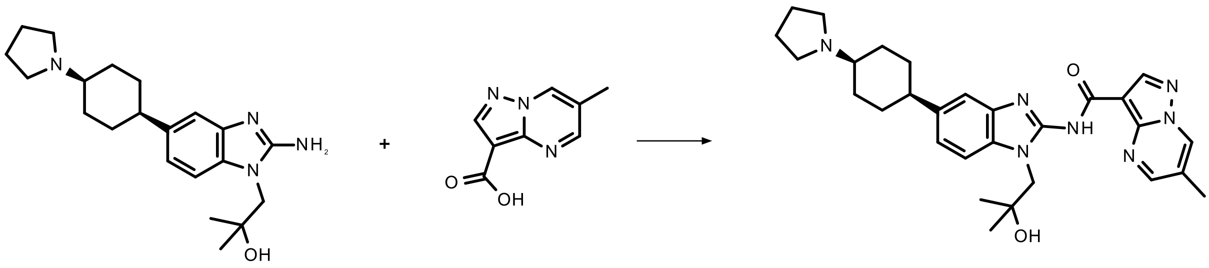

To 656 mg (3.70 mmol) 6‐methylpyrazolo[1,5‐a]pyrimidine‐3‐carboxylic acid (I.2) in 5 ml DMF was added 2.90 mL DIPEA and 1.41 g HATU. The reaction mixture was stirred for 15 min. 1.20 g (3.37 mmol) 1‐{2‐amino‐5‐[(1s,4s)‐4‐(pyrrolidin‐1‐yl)cyclohexyl]‐1H‐1,3‐benzodiazol‐1‐yl}‐2‐methylpropan‐2‐ol was diluted in 5 mL DMF and 10 mL DCM and was stirred at 50°C and was added to the first reaction mixture. The reaction mixture was stirred at 50°C over night. The reaction mixture was quenched with DCM and extracted with NaCl-solution. The organic phase was dried over Na_2SO_4 and concentrated. The residue was purified by HPLC (ACN/H_2O/TFA gradient) to get 900 mg (42% yield) of the desired product.

$C_{29}H_{37}N_7O_2$	(M = 515.6 g/mol)
ESI-MS:	516 $[M+H]^+$
R_t (HPLC):	0.43 min (method C)

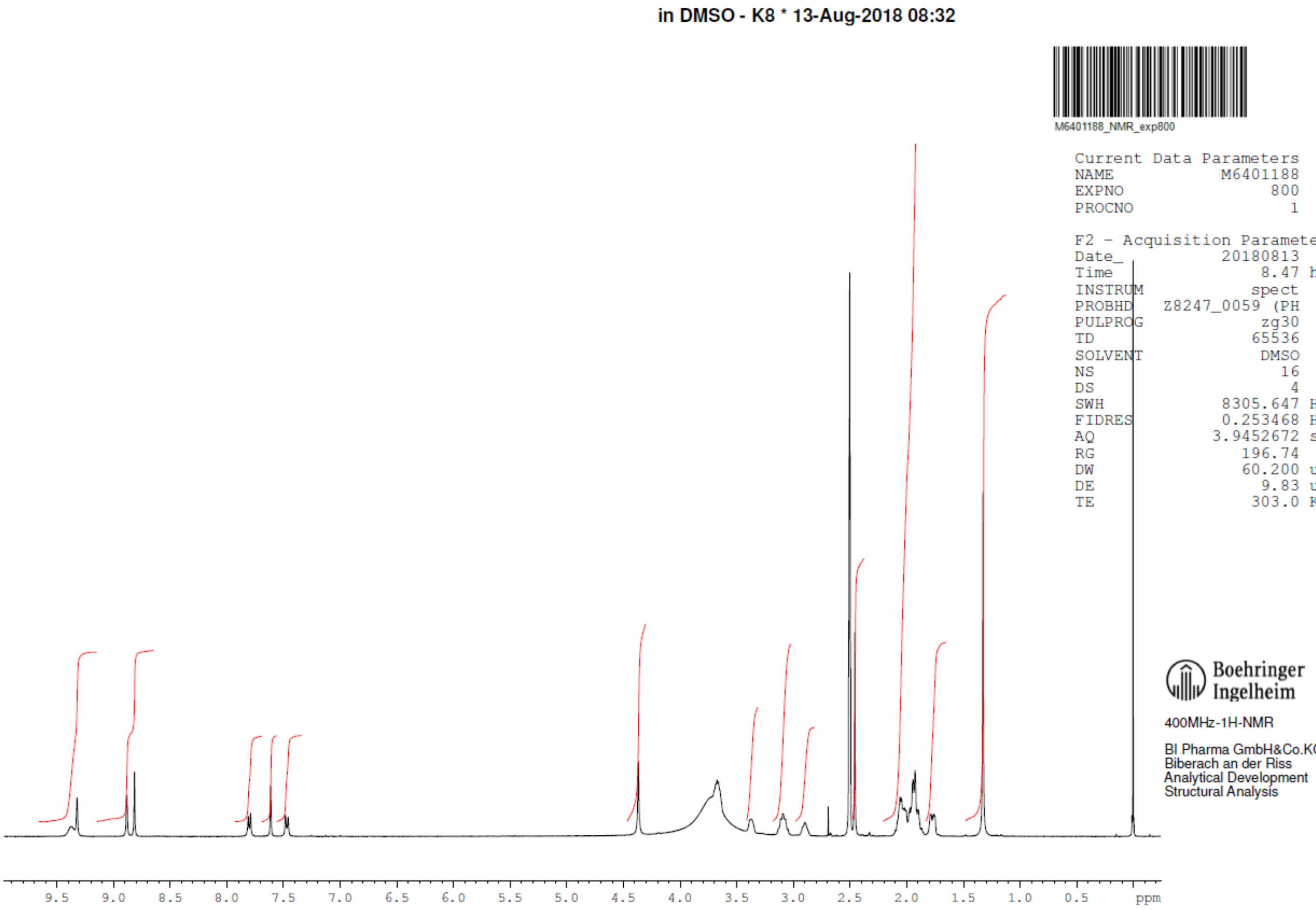

Analytical HPLC methods

Method A

time (min)	Vol% water (incl. 0.1% TFA)	Vol% ACN	Flow [mL/min]
0.00	99	1	1.6
0.02	99	1	1.6
1.00	0	100	1.6
1.10	0	100	1.6

Analytical column: XBridge BEH C18_2.1 x 30 mm, 1.7 µm; column temperature: 60°C

Method B

time (min)	Vol% water (incl. 0.1% NH_3)	Vol% ACN	Flow [mL/min]
0.00	95	5	1.3
0.02	95	5	1.3
1.00	0	100	1.3
1.10	0	100	1.3

Analytical column: XBridge BEH C18_2.1 x 30 mm, 2.5 µm; column temperature: 60°C

Method C

time (min)	Vol.% water (incl. 0.1 % TFA)	Vol. % ACN	Flow [mL/min]
0.00	95	5	1.5
1.30	0	100	1.5
1.50	0	100	1.5

Analytical column: Sunfire C18 (Waters) 2.5 µm; 3.0 x 30 mm; column temperature:

60 °C

Method D

time (min)	Vol% water (incl. 0.1% NH_3)	Vol% ACN	Flow [mL/min]
0.00	95	5	1.3

0.02	95	5	1.3
1.00	0	100	1.3
1.30	0	100	1.3

Analytical column: XBridge BEH C18_2.1 x 30 mm, 2.5 µm; column temperature: 60°C

Supplementary Table 1

Kinase selectivity of BI1543673 in the Adapta Kinase profiling Screen (Invitrogen/ThermoFisher)

**Kinase**	**% Inhibition at 10 µM**
AAK1@IG	12.0
ABL1	9.0
ABL2	4.0
ACVR1	0.0
ACVR1B	4.0
ACVR2A@IG	0.0
ACVR2B	-1.0
ACVRL1 (ALK1)@IG	3.0
ADCK3@IG	9.0
ADRBK1	5.0
ADRBK2	1.0
AKT1	4.0
AKT2	1.0

AKT3	12.0
ALK	1.0
AMPK (A1/B1/G2)@IG	-5.0
AMPK (A1/B1/G3)@IG	-1.0
AMPK (A1/B2/G1)@IG	-2.0
AMPK (A1/B2/G3)@IG	8.0
AMPK (A2/B1/G2)@IG	6.0
AMPK (A2/B1/G3)@IG	8.0
AMPK (A2/B2/G1)@IG	-3.0
AMPK (A2/B2/G2)@IG	-4.0
AMPK (A2/B2/G3)@IG	2.0
AMPK A1B1G1	-1.0
AMPK A2B1G1	14.0
AURKB	2.0
AURKC	15.0
AXL	-3.0
BLK	5.0
BMPR1A(ALK3)	-3.0
BMPR2@IG	0.0
BMX	12.0
BRAF	-3.0
BRSK1	12.0
BRSK2@IG	-7.0
BTK	7.0

CAMK1_A	8.0
CAMK1D	3.0
CAMK1G@IG	8.0
CAMK2A	-6.0
CAMK2B	9.0
CAMK2D	9.0
CAMK2G (CAMKII GA@IG	-9.0
CAMK4	-18.0
CAMKK1	-11.0
CAMKK2	2.0
CASK@IG	9.0
CDC42 BPA	5.0
CDC42 BPB	7.0
CDC42 BPG (MRCKG)@IG	3.0
CDC7/DBF4@IG	-4.0
CDK1/CYCLIN A2@IG	-17.0
CDK1/CYCLINB	5.0
CDK11 (INACTIVE)@IG	3.0
CDK14 (PFTK1)/CYC@IG	-1.0
CDK16 (PCTK1)/CYC@IG	-2.0
CDK17/CYCLIN Y@IG	9.0
CDK18/CYCLIN Y@IG	6.0

CDK2/CYCLIN A1@IG	4.0
CDK2/CYCLIN E1@IG	-4.0
CDK2/CYCLIN O@IG	-8.0
CDK2/CYCLINA	-5.0
CDK3/CYCLIN E1@IG	-5.0
CDK5 (INACTIVE)@IG	-8.0
CDK5/P25	5.0
CDK5/P35	4.0
CDK7CYCLIN	-5.0
CDK8_CYCLINC	-5.0
CDK9 (INACTIVE)@IG	-1.0
CDK9_CYCLINK	1.0
CDK9CYCLIN	12.0
CDKL5@IG	14.0
CHEK1	0.0
CHEK2	4.0
CHUK_IKKA	3.0
CLK1	20.0
CLK2	15.0
CLK3	6.0
CLK4	28.0
CSF1R	14.0
CSK	6.0
CSNK1A1	-17.0

CSNK1A1L@IG	0.0
CSNK1D	1.0
CSNK1E	-1.0
CSNK1G1	2.0
CSNK1G2	0.0
CSNK1G3	6.0
CSNK2A1	6.0
CSNK2A2	5.0
DAPK1	10.0
DAPK2@IG	-5.0
DAPK3	3.0
DCAMKL1 (DCLK1)@IG	6.0
DCAMKL2	6.0
DDR1	1.0
DDR2	1.0
DMPK	6.0
DNA-PK	8.0
DYRK1A	3.0
DYRK1B	3.0
DYRK2@IG	-2.0
DYRK3	-15.0
DYRK4	-6.0
EEF2K	11.0
EGFR	-12.0

EIF2AK2@IG	-2.0
EPHA1	12.0
EPHA2	3.0
EPHA3	-4.0
EPHA4	5.0
EPHA5	1.0
EPHA6@IG	-3.0
EPHA7	-8.0
EPHA8	1.0
EPHB1	6.0
EPHB2	4.0
EPHB3	5.0
EPHB4	2.0
ERBB2	-5.0
ERBB4	3.0
ERN1@IG	-10.0
ERN2@IG	1.0
FER	6.0
FES	-2.0
FGFR1	-8.0
FGFR2	2.0
FGFR3	0.0
FGFR4	7.0
FGR	4.0

FLT1	10.0
FLT3	33.0
FLT3 ITD@IG	45.0
FLT4	3.0
FRAP1 (MTOR)	4.0
FRK	9.0
FYN	15.0
FYN A@IG	3.0
GAK@IG	3.0
GRK1@IG	3.0
GRK4	1.0
GRK5	-4.0
GRK6	0.0
GRK7	1.0
GSG2	-2.0
GSK3A	11.0
GSK3B	3.0
HCK	3.0
HIPK1	1.0
HIPK2	0.0
HIPK3	4.0
HIPK4	2.0
HUNK@IG	0.0
ICK@IG	-3.0

IGF1R	15.0
IKBKB	0.0
IKBKE	3.0
INSR	-1.0
INSRR	10.0
IRAK1	61.0
IRAK3@IG	72.0
IRAK4	99.0
ITK	5.0
JAK1	-3.0
JAK2	-10.0
JAK2 JH1 JH2	-2.0
JAK3	-10.0
KDR	-11.0
KIT	4.0
KSR2@IG	2.0
LATS1@IG	-7.0
LATS2@IG	13.0
LCK	10.0
LIMK1	0.0
LIMK2	6.0
LRRK2	-2.0
LRRK2_FL	0.0
LTK	6.0

LYN A	1.0
LYN B	16.0
MAP2K1	-13.0
MAP2K2	-4.0
MAP2K4@IG	-8.0
MAP2K5@IG	-1.0
MAP2K6	7.0
MAP3K10	2.0
MAP3K11	7.0
MAP3K14	-2.0
MAP3K2	-1.0
MAP3K3	-8.0
MAP3K5	2.0
MAP3K7_K7IP1	21.0
MAP3K8	11.0
MAP3K9	6.0
MAP4K1 (HPK1)@IG	2.0
MAP4K2	1.0
MAP4K3 (GLK)@IG	0.0
MAP4K4	6.0
MAP4K5	6.0
MAPK1	8.0
MAPK10	13.0
MAPK11	10.0

MAPK12	16.0
MAPK13	4.0
MAPK14	8.0
MAPK14P38A	8.0
MAPK15 (ERK7)@IG	-6.0
MAPK3	3.0
MAPK7 (ERK5)@IG	5.0
MAPK8	-3.0
MAPK9	9.0
MAPKAPK2	-15.0
MAPKAPK3	-3.0
MAPKAPK5	11.0
MARK1	6.0
MARK2	12.0
MARK3@CP	6.0
MARK4	-3.0
MASTL@IG	17.0
MATK	0.0
MELK	6.0
MERTK	5.0
MET	2.0
MINK1	9.0
MKNK1	-3.0
MKNK2 (MNK2)	20.0

MLCK	-1.0
MLK4@IG	-1.0
MST1R	-14.0
MST4	9.0
MUSK	3.0
MYLK	-6.0
MYLK2	-1.0
MYLK4@IG	8.0
MYO3A@IG	-3.0
MYO3B (MYO3 BETA)@IG	11.0
NEK1	-1.0
NEK2	0.0
NEK4	3.0
NEK6	3.0
NEK7	19.0
NEK8@IG	-3.0
NEK9	3.0
NIM1K@IG	11.0
NLK	0.0
NTRK1	75.0
NTRK2	28.0
NTRK3	43.0
NUAK1	-6.0

NUAK2@IG	12.0
PAK1	5.0
PAK2	6.0
PAK3	12.0
PAK4	0.0
PAK6	3.0
PAK7	6.0
PASK	3.0
PDGFRA	-12.0
PDGFRB	19.0
PDK1	5.0
PDK1DIRECT	6.0
PEAK1@IG	13.0
PHKG1	12.0
PHKG2	6.0
PI4K2A@IG	-5.0
PI4K2B@IG	-1.0
PI4KA	-2.0
PI4KB	-11.0
PIK3C2A	-9.0
PIK3C2B	10.0
PIK3C2G@IG	-5.0
PIK3C3	-2.0
PIK3CA/PIK3R3 @IG	10.0

PIK3CAE545KPIK3R1@IG	-2.0
PIK3CAPIK3R1	-2.0
PIK3CB/PIK3R1	7.0
PIK3CB/PIK3R2@IG	-7.0
PIK3CBPIK3RI	7.0
PIK3CDPIK3R1	-10.0
PIK3CG	2.0
PIM1	6.0
PIM2	9.0
PIM3@IG	2.0
PKMYT1@IG	0.0
PKN1	-1.0
PKN2@IG	14.0
PLK1	4.0
PLK2	0.0
PLK3	7.0
PLK4@IG	-2.0
PRKACA	4.0
PRKACB (PRKAC BET@IG	7.0
PRKACG (PRKAC GAM@IG	6.0
PRKCA	15.0
PRKCB1	10.0

PRKCB2	2.0
PRKCD	10.0
PRKCE	17.0
PRKCG	13.0
PRKCH	12.0
PRKCI	7.0
PRKCN	8.0
PRKCQ	16.0
PRKCZ	19.0
PRKD1	23.0
PRKD2	3.0
PRKG1	2.0
PRKG2	9.0
PRKX	7.0
PTK2	5.0
PTK2B	3.0
PTK6	4.0
RAF1	-12.0
RET	4.0
RIPK2	16.0
RIPK3@IG	14.0
ROCK1	10.0
ROCK2	0.0
ROS1	2.0

RPS6KA1	8.0
RPS6KA2	19.0
RPS6KA3	1.0
RPS6KA4	3.0
RPS6KA5	0.0
RPS6KA6	5.0
RPS6KB1	-8.0
RPS6KB2 (P70S6KB)@IG	7.0
SBK1@IG	4.0
SGK	10.0
SGK2	5.0
SGKL	9.0
SIK1@IG	-7.0
SIK3@IG	3.0
SLK	-2.0
SNF1LK2	16.0
SPHK1	-3.0
SPHK2	-9.0
SRC	10.0
SRC N1	5.0
SRMS	10.0
SRPK1	5.0
SRPK2	3.0
STK16	-5.0

STK17A	7.0
STK17B (DRAK2)@IG	7.0
STK22B	16.0
STK22D	7.0
STK23	0.0
STK24	10.0
STK25	6.0
STK3	-3.0
STK32B (YANK2)@IG	9.0
STK32C (YANK3)@IG	-10.0
STK33	1.0
STK38 (NDR)@IG	-2.0
STK38L (NDR2)@IG	8.0
STK39 (STLK3)@IG	-4.0
STK4	11.0
STK6	3.0
SYK	4.0
TAOK1@IG	-1.0
TAOK2	10.0
TAOK3	9.0
TBK1	6.0
TEC	-1.0
TEK	-4.0
TESK1@IG	29.0

TGFBR1	-2.0
TGFBR2@IG	12.0
TLK1@IG	2.0
TLK2@IG	4.0
TNIK@IG	12.0
TNK1@IG	7.0
TNK2	-9.0
TTK	5.0
TXK	10.0
TYK2	3.0
TYRO3	9.0
ULK1@IG	-2.0
ULK2@IG	-5.0
ULK3@IG	-4.0
VRK2@IG	-8.0
WEE1	17.0
WNK2	-6.0
WNK3@IG	1.0
YES1	14.0
ZAK	-1.0
ZAP70	4.0

Supplementary Table 2

BALF cell count/ml (mean ± SEM)

Treatments	Total cell count (10^6)	Mono (10^6)	Neutro (10^6)	Lympho (10^6)	Eosino (10^6)
Sham + vehicle	0.18±0.03	0.16±0.03	0.01±0.00	0.00±0.00	0.00±0.00
LPS + vehicle	0.63±0.05*	0.08±0.01*	0.53±0.06*	0.01±0.01	0.00±0.00
LPS + 30 mg/kg	0.79±0.15#	0.08±0.01	0.69±0.13#	0.01±0.01	0.01±0.01
LPS + 100 mg/kg	0.40±0.04#	0.06±0.02	0.31±0.04#	0.00±0.00	0.02±0.01#
LPS + 300 mg/kg	0.23±0.06#	0.06±0.01	0.16±0.05#	0.00±0.00	0.00±0.00

* P<0.05 LPS + vehicle vs sham + vehicle

# P<0.05 LPS + BI1543673 vs LPS + sham

Supplementary Table 3

BALF percent of each cell type (mean ± SEM)

Treatments	Mono (%)	Neutro (%)	Lympho (%)	Eosino (%)
Sham + vehicle	89.93±1.88	8.50±1.86	1.58±2.36	0.00±0.00
LPS + vehicle	12.93±1.13*	84.54±1.32*	1.19±0.91	1.34±0.23
LPS + 30 mg/kg	10.54±0.58	87.36±0.74	1.14±0.36	0.69±0.36
LPS + 100 mg/kg	16.14±1.49	78.34±1.40#	0.37±0.88	5.03±0.53#
LPS + 300 mg/kg	29.41±1.59#	69.06±1.59#	1.06±1.59	0.46±0.46

* P<0.05 LPS + vehicle vs sham + vehicle

# P<0.05 LPS + BI1543673 vs LPS + sham
